# Rapid assessment of Watson–Crick to Hoogsteen exchange in unlabeled DNA duplexes using high-power SELOPE imino ^1^H CEST

**DOI:** 10.5194/mr-2-715-2021

**Published:** 2021-09-14

**Authors:** Bei Liu, Atul Rangadurai, Honglue Shi, Hashim M. Al-Hashimi

**Affiliations:** 1 Department of Biochemistry, Duke University School of Medicine, Durham, NC, USA; 2 Department of Chemistry, Duke University, Durham, NC, USA

## Abstract

In duplex DNA, Watson–Crick A–T and G–C base pairs (bp's) exist in dynamic equilibrium with an alternative Hoogsteen conformation, which is low in abundance and short-lived. Measuring how the Hoogsteen dynamics varies across different DNA sequences, structural contexts and physiological conditions is key for identifying potential Hoogsteen hot spots and for understanding the potential roles of Hoogsteen base pairs in DNA recognition and repair. However, such studies are hampered by the need to prepare ${}^{13}$C or ${}^{15}$N isotopically enriched DNA samples for NMR relaxation dispersion (RD) experiments. Here, using SELective Optimized Proton Experiments (SELOPE) ${}^{1}$H CEST experiments employing high-power radiofrequency fields ($B_{1}$ > 250 Hz) targeting imino protons, we demonstrate accurate and robust characterization of Watson–Crick to Hoogsteen exchange, without the need for isotopic enrichment of the DNA. For 13 residues in three DNA duplexes under different temperature and pH conditions, the exchange parameters deduced from high-power imino ${}^{1}$H CEST were in very good agreement with counterparts measured using off-resonance ${}^{13}$C / ${}^{15}$N spin relaxation in the rotating frame ($R_{1\rho }$). It is shown that ${}^{1}$H–${}^{1}$H NOE effects which typically introduce artifacts in ${}^{1}$H-based measurements of chemical exchange can be effectively suppressed by selective excitation, provided that the relaxation delay is short (≤ 100 ms). The ${}^{1}$H CEST experiment can be performed with ∼ 10× higher throughput and ∼ 100× lower cost relative to ${}^{13}$C / ${}^{15}$N $R_{1\rho }$ and enabled Hoogsteen chemical exchange measurements undetectable by $R_{1\rho }$. The results reveal an increased propensity to form Hoogsteen bp's near terminal ends and a diminished propensity within A-tract motifs. The ${}^{1}$H CEST experiment provides a basis for rapidly screening Hoogsteen breathing in duplex DNA, enabling identification of unusual motifs for more in-depth characterization.

## 1 Introduction

**Figure 1:**
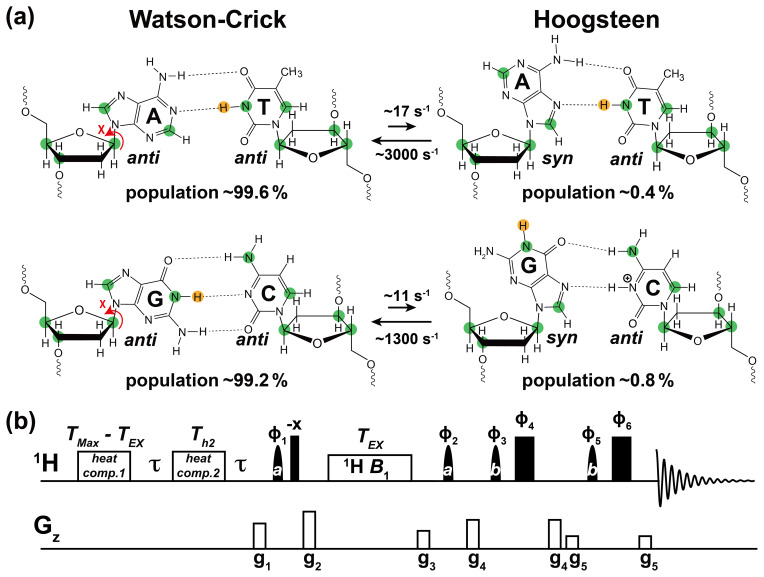
Using ${}^{1}$H CEST to measure Watson–Crick to Hoogsteen exchange in unlabeled nucleic acid duplexes. **(a)** Watson–Crick G–C and A–T bp's in B-DNA exist in dynamic equilibrium with G–C${}^{+}$ and A–T Hoogsteen bp's, respectively. Filled green circles denote nuclei (${}^{13}$C and ${}^{15}$N) that have previously been used to probe the Watson–Crick to Hoogsteen exchange via RD measurements, while the yellow circle denotes the imino ${}^{1}$H probes used in this study. Rate constants and populations were obtained as described previously (Alvey et al., 2014). **(b)** The 1D SELOPE ${}^{1}$H CEST pulse sequence for characterizing chemical exchange in unlabeled nucleic acids. Narrow and wide filled rectangles denote 90 and 180${}^{\circ }$ hard pulses. Semi-oval shapes denote selective pulses. Pulse a is a 90${}^{\circ }$ Eburp2.1000 shape pulse (typically 3–4 ms) for selective excitation (excitation bandwidth ∼ 2–3 ppm) of imino protons, while pulse b is a 180${}^{\circ }$ Squa100.1000 shape pulse with length 2 ms in an excitation sculpting scheme (Hwang and Shaka, 1995) for water suppression. Open rectangles denote the gradients and heat compensation elements. Delay $\tau =1/2d_{1}=0.7$ s. To ensure uniform heating for experiments with variable lengths of $T_{\mathrm{EX}}$, the relaxation period during which a ${}^{1}$H $B_{1}$ field is applied, two heat compensation modules were used according to a prior study (Schlagnitweit et al., 2018). The first heat compensation is applied far off-resonance with duration = $T_{\mathrm{Max}}-T_{\mathrm{EX}}=2$ ms, where $T_{\mathrm{Max}}$ is the maximum relaxation delay time. The second heat compensation (1 kHz) applied far off-resonance has a duration $T_{h2}=150$ ms. The phase cycles used are $\phi_{1}=\{8x,8(-x)\}$, $\phi_{2}=\{4x,4(-x)\}$, $\phi_{3}=\{x,y\}$, $\phi_{4}=\{-x,-y\}$, $\phi_{5}=\{2x,2y\}$, and $\phi_{6}=\{2(-x),2(-y)\}$. Gradients (g1–g5) with SMSQ10.100 profiles are applied for 1 ms with the following amplitudes (G cm${}^{-1}$): 14.445, 26.215, 14.445, 16.585, 5.885. The ${}^{1}$H carrier is placed far offset (100 000 Hz) during the two heat compensation periods, then moved to the center of the imino resonances prior to the first pulse a. Next, the carrier is placed to a specified offset prior to the relaxation delay ($T_{\mathrm{EX}}$), then placed back to the center of the imino resonances following $T_{\mathrm{EX}}$. Finally, it is placed on-resonance with water for water suppression prior to pulse b. Briefly, imino ${}^{1}$H magnetization is selectively excited, aligned longitudinally and then relaxes under a ${}^{1}$H $B_{1}$ field during $T_{\mathrm{EX}}$. ${}^{1}$H transverse magnetization is then created and directly detected following water suppression. This pulse sequence is adapted from Schlagnitweit et al. (2018).

Soon after the discovery of the DNA double helix, it was shown that A–T and G–C could also pair in an alternative conformation known as the “Hoogsteen” base pair (bp) (Felsenfeld et al., 1957; Hoogsteen, 1959) (Fig. 1a). Starting from a canonical Watson–Crick G–C or A–T bp, the corresponding Hoogsteen bp's can be obtained by flipping the purine base 180${}^{\circ }$ and bringing the two bases into proximity to create a new set of hydrogen bonds, which in the case of G–C bp's require protonation of cytosine N3 (Fig. 1a).

Following their discovery, Hoogsteen bp's were observed in crystal structures of duplex DNA in complex with proteins (Kitayner et al., 2010; Aishima et al., 2002) and drugs (Wang et al., 1984; Ughetto et al., 1985) and shown to play a role in DNA recognition (Golovenko et al., 2018), damage induction (Xu et al., 2020), and repair (Lu et al., 2010), and in damage bypass during replication (Nair et al., 2006; Ling et al., 2003). NMR relaxation dispersion (RD) studies employing off-resonance ${}^{13}$C and ${}^{15}$N spin relaxation in the rotating frame ($R_{1\rho }$) later showed that the G–C and A–T Watson–Crick bp's exist in a dynamic equilibrium with their Hoogsteen counterparts (Nikolova et al., 2011). The Hoogsteen bp's were shown to be lowly populated (population < 1 %) and short-lived (lifetime ∼ 1 ms) forming robustly as an excited conformational state (ES) in duplex DNA across a variety of sequence contexts (Alvey et al., 2014) (Fig. 1a).

There is growing interest in mapping the Watson–Crick to Hoogsteen exchange landscape across different DNA contexts, including for bp's in different sequence motifs (Alvey et al., 2014), near sites of damage and mismatches (Shi et al., 2021; Singh et al., 1993), and when DNA is bound to proteins (Nikolova et al., 2013b; Zhou et al., 2019) and drugs (Xu et al., 2018; Wang et al., 1984). Studies suggest an increased propensity to form Hoogsteen bp's in such environments (Shi et al., 2021), and this may in turn play a role in DNA recognition and damage repair (Afek et al., 2020). Furthermore, there is interest in understanding how the Hoogsteen exchange varies with temperature (Nikolova et al., 2011), pH (Nikolova et al., 2013a), salt concentration and buffer composition (Rangadurai et al., 2020b; Tateishi-Karimata et al., 2014), as well as in the presence of epigenetic modifications (Wang et al., 2017; Rangadurai et al., 2019a), all of which could shape these dynamics and consequently DNA biochemical transactions.

There are hundreds and thousands of motifs and conditions for which characterization of Hoogsteen dynamics is of biological interest. However, current approaches for measuring Hoogsteen dynamics are ill-suited for dynamics measurements at such a scale. The Watson–Crick to Hoogsteen chemical exchange process has been characterized with the use of ${}^{13}$C (Nikolova et al., 2011; Shi et al., 2018; Ben Imeddourene et al., 2020; Alvey et al., 2014) and ${}^{15}$N (Nikolova et al., 2012a; Rangadurai et al., 2019a; Alvey et al., 2014) off-resonance $R_{1\rho }$, and more recently chemical exchange saturation transfer (CEST) experiments (Rangadurai et al., 2020b, a). However, these approaches require isotopically enriched DNA samples, making broad explorations of Hoogsteen exchange across even tens of motifs impractical. Furthermore, many motifs of interest involve damaged or modified nucleotides, which are difficult to isotopically enrich with ${}^{13}$C and ${}^{15}$N nuclei. It is therefore desirable to have more facile means to initially assess Watson–Crick to Hoogsteen exchange and to follow up with in-depth characterization for those motifs exhibiting interesting and unusual behavior. For such an initial screening application, we turned our attention to the imino ${}^{1}$H as a probe of the Watson–Crick to Hoogsteen exchange in unlabeled DNA samples.

The utility of protons as probes in CEST (Chen et al., 2016; Dubini et al., 2020; Wang et al., 2021; Liu et al., 2021a), Carr–Purcell–Meiboom–Gill (CPMG) (Juen et al., 2016; LeBlanc et al., 2018), and off-resonance $R_{1\rho }$ experiments (Wang and Ikuta, 1989; Lane et al., 1993; Steiner et al., 2016; Schlagnitweit et al., 2018; Baronti et al., 2020; Furukawa et al., 2021) to study conformational exchange in nucleic acids is now well-established. Many of these ${}^{1}$H-based approaches use experiments originally developed to study conformational exchange in proteins (Ishima et al., 1998; Eichmuller and Skrynnikov, 2005; Lundstrom and Akke, 2005; Lundstrom et al., 2009; Otten et al., 2010; Bouvignies and Kay, 2012; Hansen et al., 2012; Weininger et al., 2012, 2013; Smith et al., 2015; Sekhar et al., 2016; Yuwen et al., 2017a, b). The ${}^{1}$H experiments permit the use of higher effective fields allowing characterization of conformational exchange faster than is possible using ${}^{13}$C or ${}^{15}$N experiments (Steiner et al., 2016; Palmer, 2014). Furthermore, the relationship between ${}^{1}$H chemical shifts and structure is reasonably well understood and has been exploited in the conformational characterization of nucleic acids (Sripakdeevong et al., 2014; Frank et al., 2013; Wang et al., 2021; Swails et al., 2015; Czernek et al., 2000; Lam and Chi, 2010).

Recently, ${}^{1}$H $R_{1\rho }$ and CEST SELective Optimized Proton Experiments (SELOPE) were developed and applied to characterize conformational exchange in unlabeled RNA (Schlagnitweit et al., 2018). The SELOPE experiment has already found several applications in studies of unlabeled nucleic acids, including in the characterization of fast ($k_{\mathrm{ex}}=k_{1}+k_{-1}>1000$ s${}^{-1}$) RNA secondary structural rearrangements (Baronti et al., 2020) and DNA base opening (Furukawa et al., 2021), as well as slower ($k_{\mathrm{ex}}$ < 100 s${}^{-1}$) DNA hybridization kinetics (Dubini et al., 2020). Many ${}^{1}$H relaxation dispersion (RD) studies have targeted exchangeable imino protons (Baronti et al., 2020; Furukawa et al., 2021), taking advantage of the well-known dependence of the imino ${}^{1}$H chemical shifts on secondary structure (Wang et al., 2021; Lam and Chi, 2010).

Although ${}^{1}$H RD experiments can obviate the need for isotopic labeling and offer other advantages such as high sensitivity, they have not been as widely used compared to ${}^{13}$C / ${}^{15}$N RD experiments. One reason for this has to do with potential artifacts arising due to from ${}^{1}$H–${}^{1}$H cross-relaxation (Ishima et al., 1998; Eichmuller and Skrynnikov, 2005; Lundstrom and Akke, 2005; Bouvignies and Kay, 2012). Interestingly, in nucleic acids, such NOE effects appear to be effectively suppressed in the ${}^{1}$H SELOPE experiment through selective excitation of spins (Schlagnitweit et al., 2018). The exchange parameters obtained using ${}^{1}$H SELOPE experiments were shown to be in very good agreement with counterparts obtained using ${}^{13}$C and ${}^{15}$N off-resonance $R_{1\rho }$ (Baronti et al., 2020). In addition, similar exchange parameters were obtained when using variable tilt angles in $R_{1\rho }$ experiments, including tilt angle of 35.3${}^{\circ }$ in which ROE and NOE cross-relaxation terms cancel (Eichmuller and Skrynnikov, 2005; Weininger et al., 2013; Steiner et al., 2016). No NOE dips or artifacts were observed in the majority of the ${}^{1}$H CEST or off-resonance $R_{1\rho }$ profiles (Steiner et al., 2016; Dubini et al., 2020; Furukawa et al., 2021). These results are consistent with a prior off-resonance ${}^{1}$H $R_{1\rho }$ studies showing that even without deuteration, it is feasible to effectively suppress cross-relaxation between amide and aliphatic protons through selective inversion of amide protons and use of short spin-lock relaxation delays (Lundstrom and Akke, 2005; Schlagnitweit et al., 2018). Nevertheless, NOE effects have been reported for select sites in ${}^{1}$H SELOPE studies of nucleic acids (Schlagnitweit et al., 2018) and in ${}^{1}$H CEST studies of proteins (Bouvignies and Kay, 2012; Sekhar et al., 2016; Yuwen et al., 2017a, b). This underscores the need to carefully analyze NOE effects, especially for unlabeled samples, in which spin-state-selective magnetization transfer schemes (Yuwen et al., 2017a, b) employing heteronuclei to suppress NOE effects are not feasible.

There are certain conditions in which the Hoogsteen bp becomes the dominant conformation in duplex DNA. These include chemically modified bases (Nikolova et al., 2011), when DNA is in complex with binding partners (Xu et al., 2018), and for specific sequence contexts under certain experimental conditions (Stelling et al., 2017). Based on NMR studies of such duplexes containing Hoogsteen bp's, there should be a sizable difference (Δω ∼ -1 to -2 ppm) between the imino proton chemical shifts of guanine (G-H1) and thymine (T-H3) in the Hoogsteen versus Watson–Crick conformation. These differences should render G-H1 and T-H3 suitable probes of Hoogsteen exchange in unlabeled DNA duplexes provided that NOE effects can be effectively suppressed. Imino protons are also attractive probes given that they are often well-resolved even in 1D ${}^{1}$H spectra of large RNAs. Since no other ESs have been detected to date in several NMR studies of unmodified canonical DNA duplexes (Nikolova et al., 2011; Alvey et al., 2014; Shi et al., 2018; Ben Imeddourene et al., 2020), a single imino ${}^{1}$H probe could be sufficient to reliably map and characterize the Watson–Crick to Hoogsteen exchange.

Here, we show that high-power ${}^{1}$H CEST SELOPE experiments targeting the imino protons G-H1 and T-H3 provide facile means for initially assessing Watson–Crick to Hoogsteen exchange of G–C and A–T bp's in DNA without the need for isotopic enrichment. NOE effects are shown to have a negligible contribution as short (≤ 100 ms) relaxation delays can be used to characterize the relatively fast ($k_{\mathrm{ex}}$ ∼ 500 to 8000 s${}^{-1}$) Watson–Crick to Hoogsteen exchange process (Alvey et al., 2014). The approach also takes advantage of high-power radio-frequency (RF) fields recently shown (Rangadurai et al., 2020a) to extend the timescale sensitivity of CEST to include faster exchange processes that traditionally are more effectively characterized with the use of $R_{1\rho }$. The high-power ${}^{1}$H CEST experiment also enabled measurement of fast Hoogsteen exchange kinetics ($k_{\mathrm{ex}}$ > 20 000 s${}^{-1}$) inaccessible to conventional ${}^{13}$C or ${}^{15}$N off-resonance $R_{1\rho }$ RD. The ${}^{1}$H CEST experiment opens the door to more comprehensively and systematically exploring how the Watson–Crick to Hoogsteen exchange process varies with sequence and structural contexts and physiological conditions of interest.

## 2 Results

### 2.1 Assessment of NOE effects

**Figure 2:**
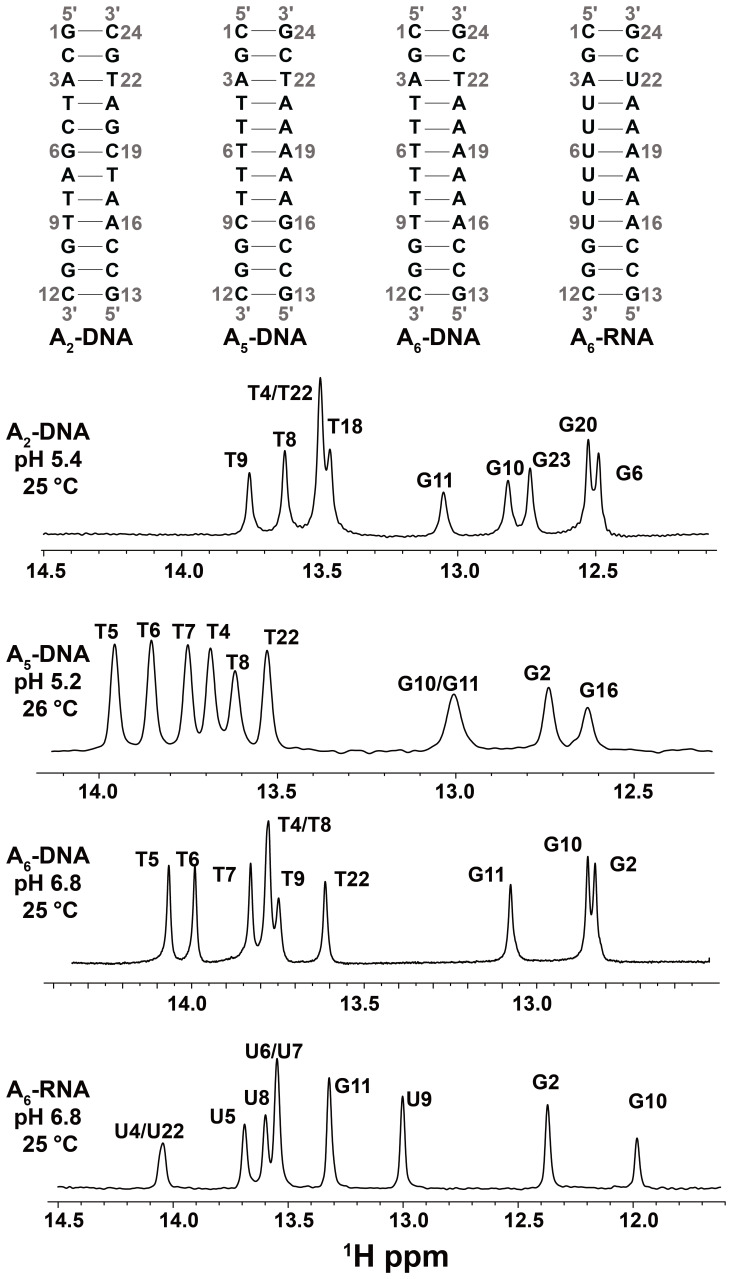
DNA and RNA duplexes used in this study. Also shown are 1D ${}^{1}$H spectra of the imino region. The buffer conditions were 25 mM sodium chloride, 15 mM sodium phosphate, 0.1 mM EDTA and 10 % D${}_{2}$O. The pH and temperature are indicated on each spectrum.

We used the SELOPE (Schlagnitweit et al., 2018) experiment (Fig. 1b) to measure ${}^{1}$H CEST profiles for G-H1 and T-H3 in unlabeled DNA duplexes (Fig. 2) at 25–26 ${}^{\circ }$C. We used ${}^{1}$H CEST rather than $R_{1\rho }$ given the greater ease of collecting profiles for many spins simultaneously, and given that with the use of high-power RF fields, CEST can effectively characterize exchange processes over a wide range of timescales (Rangadurai et al., 2020a). Use of high-power RF fields was recently shown to be important to effectively characterize the comparatively fast ($k_{\mathrm{ex}}$ ∼ 3000 s${}^{-1}$) Watson–Crick to Hoogsteen exchange process using ${}^{13}$C and ${}^{15}$N CEST experiments (Rangadurai et al., 2020a). Here, we also employed high-power RF fields (> 250 Hz) to optimally characterize Watson–Crick to Hoogsteen exchange using ${}^{1}$H CEST.

An important consideration when performing ${}^{1}$H CEST experiments is contributions due to ${}^{1}$H–${}^{1}$H cross-relaxation, which may give rise to extraneous NOE dips in the ${}^{1}$H CEST profiles (Ishima et al., 1998; Lundstrom and Akke, 2005; Eichmuller and Skrynnikov, 2005; Bouvignies and Kay, 2012; Sekhar et al., 2016; Yuwen et al., 2017a, b). These contributions have been suppressed in proteins through deuteration (Eichmuller and Skrynnikov, 2005; Lundstrom and Akke, 2005; Lundstrom et al., 2009; Otten et al., 2010; Hansen et al., 2012; Weininger et al., 2012), in ${}^{15}$N isotopically labeled proteins (Yuwen et al., 2017a, b) and nucleic acids (Wang et al., 2021; Liu et al., 2021a) using spin-state-selective magnetization transfer schemes, and through selective inversion of protons combined with use of short relaxation times (Lundstrom and Akke, 2005; Schlagnitweit et al., 2018).

In the SELOPE experiment, imino protons are selectively excited, and the magnetization belonging to non-imino protons is dephased prior to application of the $B_{1}$ field. This helps to suppress cross-relaxation (Yamazaki et al., 1994) between the imino and non-imino protons (vide infra). In addition, because the Watson–Crick to Hoogsteen exchange is relatively fast with $k_{\mathrm{ex}}$ = ∼ 500–8000 s${}^{-1}$ at 25 ${}^{\circ }$C (Alvey et al., 2014), we could afford to use a relatively short relaxation delay of 100 ms, which also helped minimize NOE effects (vide infra) (Lundstrom and Akke, 2005; Schlagnitweit et al., 2018).

**Figure 3:**
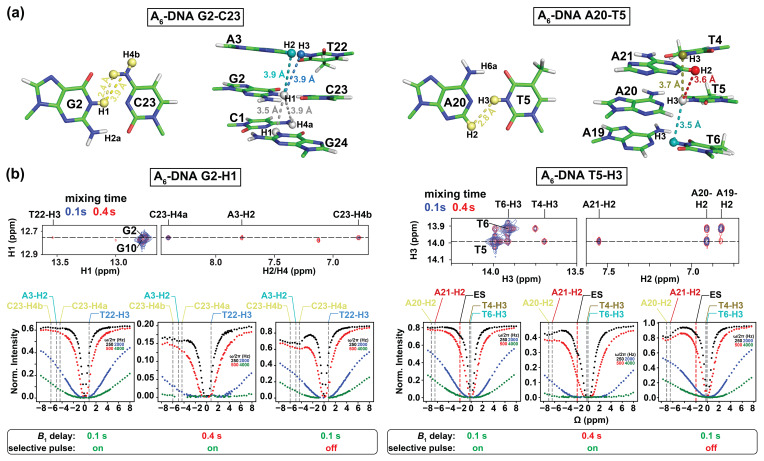
Analysis of NOE effects in ${}^{1}$H CEST profiles. **(a)** Distances between the imino protons of G2-H1 and T5-H3 and nearby protons in the A${}_{6}$-DNA duplex (PDBID: 5UZF). Note that although the amino proton of G-H2a is in proximity (2.2 Å) to G-H1, while the amino proton of A-H6a is in proximity (2.4 Å) to the partner T-H3, these amino protons are not observable in 1D ${}^{1}$H or 2D [${}^{1}$H,${}^{1}$H] NOESY spectra caused by intermediate exchange due to the restricted rotation around the C–NH${}_{2}$ bond (Schnieders et al., 2019). **(b)** NOE dips in ${}^{1}$H CEST profiles for G2-H1 and T5-H3 in A${}_{6}$-DNA. The NOE diagonal and cross peaks for G2-H and T5-H3 in the 2D [${}^{1}$H, ${}^{1}$H] NOESY spectra with mixing time 100 ms (blue) and 400 ms (red) are shown on the top. The ${}^{1}$H CEST profiles for G2-H1 and T5-H3 with combinations of short (100 ms) and long (400 ms) relaxation delays, with and without selective excitation (Methods), are shown at the bottom. The ES frequency (black) obtained from fitting ${}^{1}$H CEST profiles with selective excitation and short relaxation delay (100 ms) as well as frequency positions corresponding to the NOE cross peaks in the 2D [${}^{1}$H, ${}^{1}$H] NOESY spectra (top) are highlighted according to the color scheme in **(a)** (bottom). Error bars for CEST profiles in **(b)**, which are smaller than the data points, were obtained using triplicate experiments, as described in Methods. RF powers for CEST profiles are color-coded.

We initially performed experiments to evaluate contributions from ${}^{1}$H–${}^{1}$H cross-relaxation to the imino ${}^{1}$H CEST profiles. In canonical B-form DNA and A-form RNA duplexes (Fig. 2), G-H1 is in closest proximity to the partner base C-H4a (∼ 2.4 Å, Fig. 3a), while T/U-H3 is in closest proximity to the partner A-H2 (∼ 2.8 Å, Fig. 3a). Additional proximal protons include imino and H2 protons of neighboring residues (∼ 3.5–3.6 Å, Fig. 3a). These short internuclear distances are reflected in the intensity of cross peaks in 2D [${}^{1}$H, ${}^{1}$H] NOESY spectra of nucleic acid duplexes (Figs. 3b and S1 in the Supplement). Note that although the amino proton of G-H2a is in proximity (2.2 Å) to G-H1, while the amino proton of A-H6a is in proximity (2.4 Å) to the partner T-H3 (Fig. 3a), these amino protons are typically not observable in 1D ${}^{1}$H or 2D [${}^{1}$H,${}^{1}$H] NOESY spectra caused by intermediate exchange due to the restricted rotation around the C–NH${}_{2}$ bond (Schnieders et al., 2019).

${}^{1}$H CEST profiles (Figs. 3b and S2) for well-resolved imino resonances of A${}_{6}$-DNA (Fig. 2) were acquired simultaneously in a 1D manner using ∼ 3 h of acquisition time on a spectrometer operating at 600 MHz ${}^{1}$H frequency equipped with a cryogenic probe, using ∼ 1.0 mM unlabeled DNA (Methods). Data were initially collected at pH = 6.8. Under these near-neutral pH conditions, it is generally not feasible to detect the Watson–Crick to Hoogsteen exchange process for G–C bp's due to the low population of the protonated G–C${}^{+}$ Hoogsteen bp's (Nikolova et al., 2013a). The lack of expected dips for the ES G–C${}^{+}$ Hoogsteen bp's under these conditions provides an opportunity to better assess any extraneous ${}^{1}$H CEST dips arising due to NOE effects. Unlike for G–C bp's, the Hoogsteen exchange should still be detectable for A–T bp's under these pH conditions.

Shown in Fig. 3b is a representative imino ${}^{1}$H CEST profile measured for G2-H1 in the well-characterized A${}_{6}$-DNA duplex (Nikolova et al., 2011). Besides the major dip, no additional dips were visible in the ${}^{1}$H CEST profile. The major dip was also symmetric (Rangadurai et al., 2020a), indicating little to no contribution from Hoogsteen exchange or NOE effects, as expected for G–C bp's under these pH conditions (Nikolova et al., 2013a). On the other hand, a minor shoulder was observed in the ${}^{1}$H CEST profile of T5-H3 (Fig. 3b, the Δω is highlighted by a dashed red line and labeled “ES”). The shoulder occurs at an offset frequency that does not correspond with any other observable proton frequency in the A${}_{6}$-DNA duplex and is therefore unlikely to be the result of NOE effects (Fig. 3a). Rather, as will be described below, the shoulder corresponds to the ES Hoogsteen bp, which is to be expected for the A–T bp at pH = 6.8.

**Figure 4:**
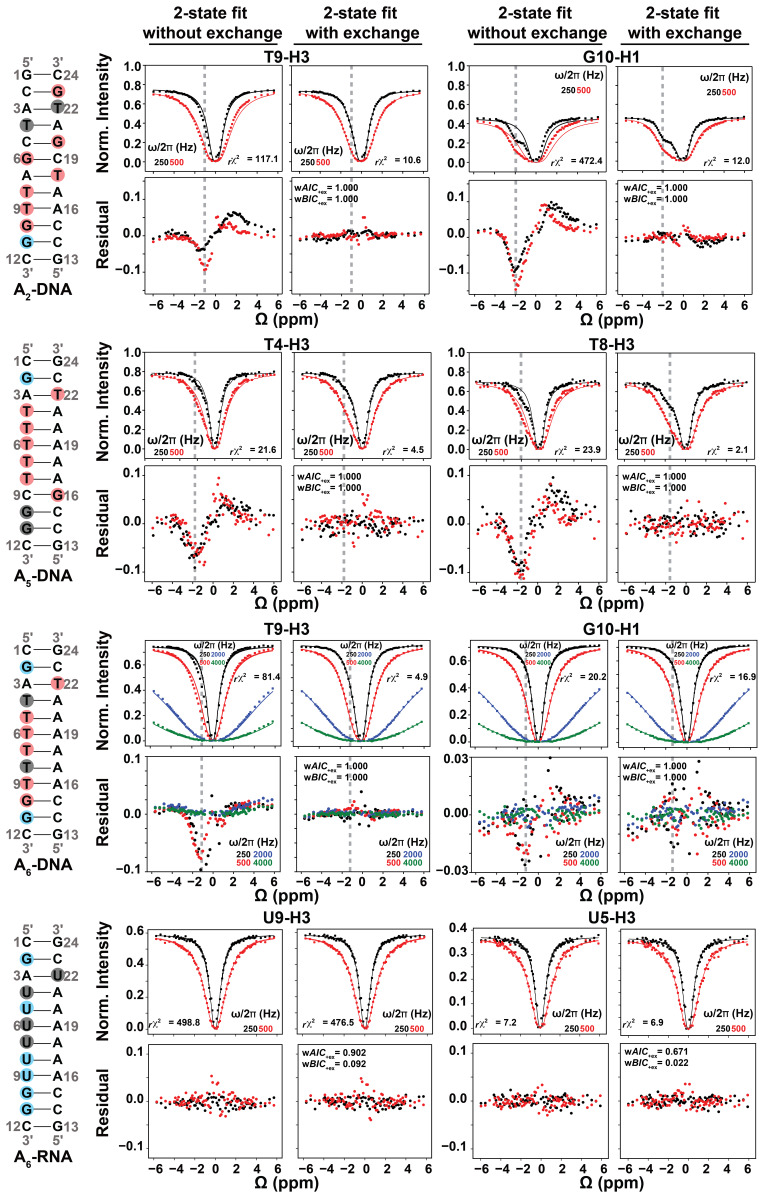
Representative ${}^{1}$H CEST profiles measured for A${}_{2}$-DNA (pH 5.4) at 25 ${}^{\circ }$C, A${}_{5}$-DNA (pH 5.2) at 26 ${}^{\circ }$C, A${}_{6}$-DNA (pH 6.8) at 25 ${}^{\circ }$C and A${}_{6}$-RNA (pH 6.8) at 25 ${}^{\circ }$C. Residues with detectable RD, undetectable RD and overlapped 1D ${}^{1}$H resonances (see Fig. 2) are highlighted in red, blue and gray circles respectively. Shown are the fits of the ${}^{1}$H CEST data to a two-state Bloch–McConnell equation with and without ($k_{\mathrm{ex}}=\Delta \omega =p_{\mathrm{ES}}=0$) chemical exchange. Shown below the CEST profiles are residual (experimental normalized intensity – fitted normalized intensity) plots. Also shown are the reduced chi-square ($r\chi^{2}$), and Akaike's (wAIC) and Bayesian information criterion (wBIC) weights for fits with exchange (Methods). The dashed gray lines indicate the Hoogsteen Δω positions in both ${}^{1}$H CEST profiles and in residual plots. Error bars for CEST profiles, which are smaller than the data points, were obtained using triplicate experiments, as described in Methods. RF powers for CEST profiles are color-coded.

To further verify that the dips observed in the ${}^{1}$H CEST profile of T5-H3 and other thymine residues in A${}_{6}$-DNA (see Figs. 4 and S2) do not represent an NOE effect, but rather reflect the ES Hoogsteen bp, we performed ${}^{1}$H CEST experiments on a corresponding A${}_{6}$-RNA duplex (Fig. 2). Unlike in B-form DNA duplexes, G–C${}^{+}$ and A–U Hoogsteen bp's are both undetectable in A-form RNA duplexes by off-resonance ${}^{13}$C and ${}^{15}$N $R_{1\rho }$ RD, most likely due their much lower population ($p_{\mathrm{ES}}$ < 0.04 %) (Zhou et al., 2016; Rangadurai et al., 2018). If the shoulder observed in the ${}^{1}$H CEST profile of T5-H3 in A${}_{6}$-DNA is due to a Hoogsteen ES, and not NOE dips, we would expect to observe a symmetric profile without ES dips for U5-H3 in A${}_{6}$-RNA. Indeed, the corresponding ${}^{1}$H CEST profiles for U5-H3 (Fig. 4) and all other uridine and guanine (Fig. S3) imino protons in A${}_{6}$-RNA were symmetric, with no evidence for any asymmetry or shoulder, indicating the absence of exchange and NOE effects.

Therefore, the shoulders in the ${}^{1}$H CEST profiles (Figs. 3, 4, S2, S3) most likely rise due to chemical exchange with an ES. This was further confirmed by evaluating whether fits to the ${}^{1}$H CEST profiles show any statistically significant improvement with the inclusion of exchange, as described below. Based on a similar analysis, no NOE dips were observable in the ${}^{1}$H CEST profiles (Figs. 4, S2, S3) for all other residues in A${}_{6}$-DNA, A${}_{6}$-RNA, and in two other DNA duplexes across a range of pH and temperature conditions when using selective excitation and relaxation delay of 100 ms (Figs. 2, 4, S2, S3). These results indicate that any NOE effects between imino and non-imino protons are small under these experimental conditions.

Upon increasing the relaxation delay to 400 ms or using a non-selective ${}^{1}$H excitation pulse (pulse a in Fig. 1b) with a delay of 100 ms, NOE dips became visible in the ${}^{1}$H CEST profiles as shown for G2-H1 and T5-H3 (Fig. 3b) in A${}_{6}$-DNA. The dips occurred at the ${}^{1}$H resonance frequency of nearby protons and, as expected, were particularly pronounced for the partner C-H4a in the case of G2-H1 and the partner A-H2 in the case of T5-H3 (Fig. 3b). Nevertheless, even the ${}^{1}$H CEST profiles acquired with 400 ms delay could be fit when restricting the offset to the imino proton region (-3 to 3 ppm), and the fitted exchange parameters were similar to those obtained from fitting profiles with 100 ms relaxation delay in which no NOE dips were visible (Fig. S4, Table S1). In contrast, the ${}^{1}$H CEST profiles measured using non-selective excitation, which had larger NOE dips relative to using a selective excitation pulse, could not be satisfactorily fit (Fig. S4).

No NOE dips were observed at the chemical shift of imino protons belonging to neighboring residues in ${}^{1}$H CEST profiles measured in DNA and RNA duplexes, and none of the ${}^{1}$H CEST profiles collected in thus study yielded an ES with Δω compatible with the imino ${}^{1}$H chemical shift of a neighboring residue. Nevertheless, these NOE effects could be more difficult to assess given that they would be buried within the major dip. While imino–imino ${}^{1}$H NOEs are not suppressed by selective excitation, their contribution is expected to be smaller relative to other NOE dips observed when using non-selective excitation (distances ∼ 2.4–2.8 Å between guanosine/thymine imino and cytosine amino/adenine H2) due the larger distance of separation between neighboring imino protons (∼ 3.5–3.9 Å) (Fig. 3a).

To further assess the impact of imino–imino ${}^{1}$H NOEs on the ${}^{1}$H CEST profiles, we examined whether selective excitation of imino protons (but not their immediate neighbors) results in different ${}^{1}$H CEST profiles relative to an experiment in which all imino protons are excited. We performed an experiment selectively exciting G10-H1 and G2-H1 in A${}_{6}$-DNA without exciting the imino resonances belonging to either of their two immediate neighbors. Selective excitation of individual imino protons resulted in ${}^{1}$H CEST profiles (Fig. S2) and fitted parameters (Table S1) for G10-H1 and G2-H1 that are within error to those obtained when exciting all imino protons, again indicating that any imino–imino NOE contribution is negligible. Finally, the impact of imino–imino NOEs on the determination of the exchange parameters was also assessed (vide infra) through comparison of the exchange parameters derived from fitting the imino ${}^{1}$H CEST profiles with those measured independently using off-resonance ${}^{13}$C and ${}^{15}$N $R_{1\rho }$ RD measurements.

These results underscore the importance of critically evaluating the NOE contributions on a case-by-case basis (Schlagnitweit et al., 2018) and also suggest that NOE effects can be effectively suppressed for the canonical duplexes used in this study provided use of selective excitation and short relaxation delays.

It should be noted that to avoid any complexities due to NOE effects with water protons or hydrogen exchange, we restricted the offset to -6 to 6 ppm when analyzing and fitting the ${}^{1}$H CEST profiles. This is common practice as relatively narrow offsets (< 4 ppm) were used in prior ${}^{1}$H CEST studies of both nucleic acids (Dubini et al., 2020; Wang et al., 2021; Liu et al., 2021a) and proteins (Yuwen et al., 2017a, b). While we did not observe a dip near the water chemical shift in the ${}^{1}$H CEST profile for the internal residue T5-H3, a weak and broad dip near the water chemical shift was observed in the profile for the near-terminal residue G2-H1 (Fig. S2). The latter dip could be due to NOEs between G2-H1 and water protons and/or due to fast hydrogen exchange kinetics.

### 2.2 Benchmarking the utility of ${}^{1}$H CEST to probe Watson–Crick to Hoogsteen exchange in DNA duplexes

To examine the utility of the SELOPE ${}^{1}$H CEST experiment to characterize Watson–Crick to Hoogsteen exchange, we benchmarked the experiment by measuring conformational exchange in three DNA duplexes (A${}_{6}$-DNA, A${}_{2}$-DNA and A${}_{5}$-DNA, Fig. 2) for which we have previously extensively characterized the Watson–Crick to Hoogsteen exchange using ${}^{13}$C and ${}^{15}$N off-resonance $R_{1\rho }$ (Nikolova et al., 2011; Alvey et al., 2014; Shi et al., 2018) and CEST (Rangadurai et al., 2020a, b) experiments. We compared the exchange parameters derived using ${}^{1}$H CEST with counterparts derived using ${}^{13}$C / ${}^{15}$N $R_{1\rho }$ or CEST for a variety of G–C and A–T bp's across three different DNA duplexes and varying pH (5.2–6.8) conditions. All ${}^{1}$H CEST experiments were performed using 100 ms relaxation delay and selective excitation.

As expected, for several thymine residues, the imino ${}^{1}$H CEST profile was visibly asymmetric (Figs. 4, S2, S3), consistent with relatively fast ($k_{\mathrm{ex}}$ > 1000 s${}^{-1}$) Watson–Crick to Hoogsteen exchange. The asymmetry manifests as an upfield shifted shoulder (e.g., T8-H3 in A${}_{5}$-DNA in Fig. 4) as expected for T-H3 Hoogsteen chemical shift (Δω ∼ -2 ppm) (Nikolova et al., 2011; Xu et al., 2018). In other cases, such as T9-H3 in A${}_{6}$-DNA, the asymmetry was less pronounced, and the exchange contribution was only apparent following comparison of fits with and without exchange (see Fig. 4).

As expected, at pH = 6.8, the imino ${}^{1}$H CEST profiles were symmetric for most guanine residues consistent with no observable exchange (Figs. 4, S2, S3). However, the major dip became asymmetric for several guanine residues when lowering the pH to 5.2 or 5.4, as expected for the Watson–Crick to Hoogsteen exchange of G–C bp's, which is favored at lower pH (Figs. 4 and S3). All minor dips occurred at resonance frequencies that did not correspond with any other protons in the molecule (Figs. 2, S1, S2). In all cases, the ${}^{1}$H CEST profiles could be satisfactorily fit to a two-state model with or without exchange, suggesting that any NOE contribution to the ${}^{1}$H CEST profile is likely to be insignificant.

To identify which imino ${}^{1}$H CEST profiles have significant chemical exchange contributions, each profile was subjected to a fit with or without ($\Delta \omega =p_{\mathrm{ES}}=k_{\mathrm{ex}}=0$) two-state chemical exchange (Methods). Akaike information criterion (AIC) and Bayesian information criterion (BIC) (Burnham and Anderson, 2004) weights were then used to evaluate whether any improvement in the fit due to inclusion of chemical exchange was statistically significant (Kimsey et al., 2018; Liu et al., 2021a). The improvement of fit was considered to be statistically significant when both AIC and BIC weights are > 0.995 and the reduced chi-square ($r\chi^{2}$) is reduced with the inclusion of exchange. Residual plots were also used to visualize changes in fit quality (Fig. 4).

Based on the AIC and BIC analysis, all thymine and guanine residues shown previously to undergo Watson–Crick to Hoogsteen exchange using off-resonance ${}^{13}$C and/or ${}^{15}$N $R_{1\rho }$ under these experimental conditions also showed statistically significant improvements when fitting the ${}^{1}$H CEST profiles with the inclusion of chemical exchange (Figs. 4, S2, S3). On the other hand, all guanine residues, including G2 and G11 in A${}_{6}$-DNA and G11 in A${}_{2}$-DNA, which did not show signs of Hoogsteen exchange in off-resonance ${}^{13}$C and/or ${}^{15}$N $R_{1\rho }$ (Nikolova et al., 2011; Shi et al., 2018) under these experimental conditions, also did not show statistically significant improvements when fitting their ${}^{1}$H CEST profiles with the inclusion of chemical exchange (Figs. 4, S2, S3).

Interestingly, a few residues, including T5, T6, T7 and T22 in A${}_{6}$-DNA and T18, G6 and G20 in A${}_{2}$-DNA (Figs. S2, S3), showed exchange based on ${}^{1}$H CEST but did not show evidence for Hoogsteen exchange based on prior off-resonance ${}^{13}$C and/or ${}^{15}$N $R_{1\rho }$ experiments (Nikolova et al., 2011; Alvey et al., 2014; Shi et al., 2018). As will be elaborated in the following section, these data provide new insights into the Watson–Crick to Hoogsteen exchange process and suggest that at least in some cases, ${}^{1}$H CEST can exceed the detection limits of ${}^{13}$C / ${}^{15}$N-based methods.

In addition, T18 and G20 in A${}_{2}$-DNA were difficult to probe using ${}^{13}$C RD due to spectra overlap (Nikolova et al., 2011) but could easily be measured using ${}^{1}$H CEST (Figs. 2, 4 and S3). In contrast, other residues such as T8 and T4 in A${}_{6}$-DNA, T4 and T22 in A${}_{2}$-DNA, and G10 and G11 in A${}_{5}$-DNA could be targeted for ${}^{13}$C or ${}^{15}$N RD measurements (Nikolova et al., 2011; Alvey et al., 2014) but could not be measured by ${}^{1}$H CEST due to overlap in the 1D ${}^{1}$H imino spectra (Fig. 2). This highlights the complementarity of ${}^{1}$H and ${}^{13}$C / ${}^{15}$N RD in characterizing Watson–Crick to Hoogsteen exchange.

To assess how well the exchange parameters are determined by the ${}^{1}$H CEST data, we subjected the ${}^{1}$H CEST profiles for residues T7 ($k_{\mathrm{ex}}/\Delta \omega ∼$ 0.2), T9 ($k_{\mathrm{ex}}/\Delta \omega ∼$ 0.82) and T22 ($k_{\mathrm{ex}}/\Delta \omega ∼$ 3.5), which exhibit exchange on the slow, intermediate and fast timescales (Rangadurai et al., 2019b) respectively, to a degeneracy analysis. We computed the reduced chi-square ($r\chi^{2}$) for a two-state fit as a function of varying $k_{\mathrm{ex}}$, Δω or $p_{\mathrm{ES}}$. In all cases, the $r\chi^{2}$ values increased significantly (up to 10-fold) when varying $k_{\mathrm{ex}}$, Δω or $p_{\mathrm{ES}}$ by 3-fold (Fig. S5), indicating that the exchange parameters are well-defined by the ${}^{1}$H CEST data.

**Figure 5:**
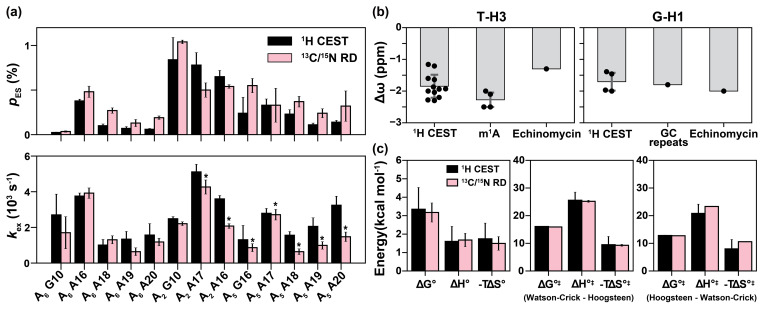
Comparison of exchange parameters for the Watson–Crick to Hoogsteen exchange obtained from ${}^{1}$H CEST and ${}^{13}$C / ${}^{15}$N $R_{1\rho }$. **(a)** Comparison of exchange parameters ($k_{\mathrm{ex}}$ and $p_{\mathrm{ES}}$) measured using ${}^{1}$H CEST with counterparts previously reported using ${}^{13}$C / ${}^{15}$N off-resonance $R_{1\rho }$ (Nikolova et al., 2011; Alvey et al., 2014; Shi et al., 2018). ${}^{13}$C RD data for A18, A19 and A20 were measured using off-resonance $R_{1\rho }$ in this study (Fig. S7). Small systematic deviations in $k_{\mathrm{ex}}$ for the values indicated with asterisks could be due to small differences in temperature (< 0.8 ${}^{\circ }$C) across different spectrometers. The bp's are specified by the corresponding purine residue. **(b)** Comparison of the Δω obtained from fitting ${}^{1}$H CEST profiles for T-H3 and G-H1 (Table S1) with the values expected for a Watson–Crick to Hoogsteen transition based on duplexes in which A–T or G–C${}^{+}$ Hoogsteen bp's were rendered the dominant state, by using $N^{1}$-methylated adenine (m${}^{1}$A) (Nikolova et al., 2011; Sathyamoorthy et al., 2017; Rangadurai et al., 2020b), by binding of the drug (echinomycin) to a DNA duplex (Xu et al., 2018), or through use of GC repeat sequences (GC repeats) that predominantly form Hoogsteen bp's at low pH (Stelling et al., 2017). **(c)** Comparison of free energy (ΔG${}^{\circ }$), enthalpy (ΔH${}^{\circ }$) and entropy (-TΔS${}^{\circ }$, T = 25 ${}^{\circ }$C) of the Watson–Crick to Hoogsteen transition, and the activation free energy (ΔG${}^{\circ }$${}^{‡}$), enthalpy (ΔH${}^{\circ }$${}^{‡}$) and entropy (-TΔS${}^{\circ }$${}^{‡}$, T = 25 ${}^{\circ }$C) for Watson–Crick to Hoogsteen (Watson–Crick–Hoogsteen) and Hoogsteen to Watson–Crick (Hoogsteen–Watson–Crick) transitions measured using ${}^{1}$H CEST in this study and using ${}^{13}$C $R_{1\rho }$ from Nikolova et al. (2011). The energetics in **(c)** were measured for the Watson–Crick to Hoogsteen transition of A16-T9 in A${}_{6}$-DNA at pH 6.8. Errors in **(a)** were fitting errors of ${}^{1}$H CEST, calculated as described in Methods or errors of ${}^{13}$C / ${}^{15}$N $R_{1\rho }$ calculated using a Monte Carlo scheme as described previously (Rangadurai et al., 2019b). Errors in **(b)** are the standard deviations of data points (shown as black dots) in each category. Error bars in **(c)** were propagated from the errors in the exchange parameters obtained from ${}^{1}$H CEST or ${}^{13}$C / ${}^{15}$N $R_{1\rho }$.

To test the accuracy of the exchange parameters obtained using ${}^{1}$H CEST, we compared the exchange parameters $p_{\mathrm{ES}}$ and $k_{\mathrm{ex}}$, derived from a two-state fit of the data to values determined previously using off-resonance ${}^{13}$C and/or ${}^{15}$N $R_{1\rho }$ (Nikolova et al., 2011; Shi et al., 2018; Alvey et al., 2014) for Hoogsteen dynamics (Fig. 5a and Table S1). In total, we were able to compare 13 data points from ${}^{1}$H CEST and ${}^{13}$C / ${}^{15}$N $R_{1\rho }$ for three different duplexes under different conditions of temperature and pH (Figs. 2, 5a). This comparison also allowed us to further verify that the exchange process detected by ${}^{1}$H CEST does indeed correspond to Watson–Crick to Hoogsteen exchange and to also further assess for potential contributions from NOE effects, which might cause deviations from agreement.

Indeed, the $p_{\mathrm{ES}}$ and $k_{\mathrm{ex}}$ values derived using ${}^{1}$H CEST were in very good agreement with their off-resonance ${}^{13}$C and/or ${}^{15}$N $R_{1\rho }$ counterparts (Fig. 5a). The differences between $k_{\mathrm{ex}}$ and $p_{\mathrm{ES}}$ measured using the two methods were often within error with the largest differences being < 3-fold. A small and systematic difference in $k_{\mathrm{ex}}$ was observed for a subset of the data (Fig. 5a), and this might be due to small temperature differences (< 0.8 ${}^{\circ }$C) between spectrometers. Importantly, the ES imino ${}^{1}$H chemical shifts deduced from a two-state fit of the ${}^{1}$H CEST profiles ($\Delta \omega_{\text{A-T}}=∼$ -1 to -2 ppm and $\Delta \omega_{\text{G-C}}=∼$ -1.5 to -2.0 ppm) were also in good agreement with the expected range of values (Δω=-1 to -2 ppm) for Hoogsteen bp's (Fig. 5b) based on studies of duplexes containing Hoogsteen bp's as the dominant conformation (Nikolova et al., 2011; Stelling et al., 2017; Xu et al., 2018; Rangadurai et al., 2020b).

As an additional test, we also measured temperature-dependent (5, 10, 20, 25, 30 and 45 ${}^{\circ }$C) ${}^{1}$H CEST profiles for A${}_{6}$-DNA at pH 6.8 (Fig. S2) and then used the temperature dependence of the fitted kinetic rate constants ($k_{1}$ and $k_{-1}$) to determine the standard and activation enthalpy and entropy changes for the Watson–Crick to Hoogsteen transition (Fig. S6). These values were in excellent agreement with those measured from off-resonance ${}^{13}$C $R_{1\rho }$ (Nikolova et al., 2011) (Fig. 5c), further supporting the robustness of the ${}^{1}$H CEST methodology.

### 2.3 New insights into Hoogsteen breathing

${}^{1}$H CEST profiles for some residues show detectable exchange contributions when corresponding ${}^{13}$C / ${}^{15}$N RD measurements do not or show only weak exchange. This suggests that ${}^{1}$H CEST can provide additional insights into Watson–Crick to Hoogsteen exchange and extend the detection limits of conventional ${}^{13}$C / ${}^{15}$N RD measurements.

For example, using ${}^{1}$H CEST it was feasible to measure Watson–Crick to Hoogsteen exchange for T5-H3, T6-H3 and T7-H3 (Fig. S2) within the middle of the A-tract motif (defined as A${}_{n}$-tract with n > 3) in A${}_{6}$-DNA. These residues had previously exhibited only weak on-resonance ${}^{13}$C $R_{1\rho }$ RD, and as a result, no off-resonance $R_{1\rho }$ data were ever recorded (Nikolova et al., 2011). Based on the ${}^{1}$H CEST measurements, residues within the A-tract motif have 10-fold lower Hoogsteen population ($p_{\mathrm{ES}}=0.06$ ± 0.01 %–0.09 ± 0.03 %) relative to other A–T bp's in A${}_{6}$-DNA ($p_{\mathrm{ES}}$ > ∼ 0.10 %) (Table S1). These represent the lowest A–T Hoogsteen populations ever recorded to date in duplex DNA (Table S1). The exchange kinetics were also 2-fold slower ($k_{\mathrm{ex}}$ ∼ 1000 s${}^{-1}$) for the A-tract residues relative to other A–T bp's ($k_{\mathrm{ex}}$ > 2000 s${}^{-1}$) in A${}_{6}$-DNA (Table S1). Interestingly, the suppression of Hoogsteen dynamics within the A-tract motif appears to be A-tract length dependent, with both the Hoogsteen population and exchange kinetics increasing slightly for similar bp's in A${}_{5}$-DNA (Table S1). The suppression of Hoogsteen dynamics within A-tracts is consistent with prior studies showing them to be more rigid and stiff motifs relative to scrambled DNA (Nikolova et al., 2012b). We verified these ${}^{1}$H CEST-derived exchange parameters for A-tract residues in A${}_{6}$-DNA by performing off-resonance ${}^{13}$C $R_{1\rho }$ measurements (Fig. S7) on uniformly ${}^{13}$C / ${}^{15}$N-labeled A${}_{6}$-DNA and did indeed observe the expected RD with $p_{\mathrm{ES}}$ and $k_{\mathrm{ex}}$ values similar (difference < 3-fold, Fig. 5a) to those measured using ${}^{1}$H CEST. These prospective tests of the ${}^{1}$H CEST data using off-resonance ${}^{13}$C / ${}^{15}$N $R_{1\rho }$ RD data further support the methodology.

The ability to characterize fast exchange kinetics has long been a motivation for using ${}^{1}$H in RD experiments to characterize conformational exchange (Ishima et al., 1998; Ishima and Torchia, 2003; Eichmuller and Skrynnikov, 2005; Lundstrom and Akke, 2005; Otten et al., 2010; Hansen et al., 2012; Smith et al., 2015; Steiner et al., 2016; Furukawa et al., 2021). Indeed, ${}^{1}$H CEST made it possible to measure fast Watson–Crick to Hoogsteen exchange kinetics which were undetectable by off-resonance ${}^{13}$C $R_{1\rho }$. In particular, it was possible to measure Watson–Crick to Hoogsteen exchange for T22 in A${}_{6}$-DNA with $k_{\mathrm{ex}}$ > 20 000 s${}^{-1}$ (Fig. S2 and Table S1), which is the fastest ever recorded Hoogsteen exchange process at 25 ${}^{\circ }$C (Table S1). In contrast, the off-resonance ${}^{13}$C $R_{1\rho }$ RD profiles reported for this residue in prior studies were flat (Nikolova et al., 2011; Shi et al., 2018), and simulations show that such an exchange process is too fast for reliable detection using ${}^{13}$C $R_{1\rho }$ (Fig. S8a). Similarly, it was feasible to measure Watson–Crick to Hoogsteen exchange for G6 ($p_{\mathrm{ES}}$ ∼ 0.3 %, $k_{\mathrm{ex}}$ ∼ 3000 s${}^{-1}$) in A${}_{2}$-DNA using ${}^{1}$H CEST, yet no off-resonance ${}^{13}$C $R_{1\rho }$ RD on C1${}^{\prime }$ was previously detected (Shi et al., 2018), which based on simulations, was likely due to a combination of exchange kinetics and small Δω value (Fig. S8b).

One of the potential utilities of the ${}^{1}$H CEST experiment is the measurement of very fast exchange kinetics at high temperatures and in a manner insensitive to melting of duplexes, shown previously to complicate analysis of Hoogsteen exchange using ${}^{13}$C and ${}^{15}$N RD (Shi et al., 2019). Melting of duplexes should not yield any exchange dips around the imino ${}^{1}$H region given that the imino protons of single-stranded species (ssDNA) exchange rapidly with solvent.

We therefore measured ${}^{1}$H CEST profiles for A${}_{6}$-DNA at 45 ${}^{\circ }$C (Fig. S2), in which the ssDNA population is ∼ 10 % (Shi et al., 2019). We did not observe any evidence for the ssDNA species in the ${}^{1}$H CEST profiles. Instead, we were able to observe ultra-fast ($k_{\mathrm{ex}}$ ∼ 10 000 s${}^{-1}$, see Table S1) Hoogsteen exchange which could not previously be detected by ${}^{13}$C or ${}^{15}$N RD experiments at the same temperature (Shi et al., 2019).

Taken together, these results demonstrate that the ${}^{1}$H CEST experiment broadens the range of populations and exchange rates over which Hoogsteen breathing can be effectively characterized.

## 3 Discussion

Building on prior studies showing the utility of the SELOPE ${}^{1}$H RD experiment in measuring conformational exchange in unlabeled RNA (Schlagnitweit et al., 2018) and DNA (Furukawa et al., 2021; Dubini et al., 2020), our study establishes the utility of high-power ${}^{1}$H CEST SELOPE as a facile means for rapidly assessing the Watson–Crick to Hoogsteen exchange process in nucleic acids without the need for isotopic enrichment. The methodology is supported by the very good agreement observed between the measured exchange parameters and values measured independently using ${}^{13}$C and/or ${}^{15}$N $R_{1\rho }$ for a variety of bp's in three duplexes under different conditions of temperature and pH, as well as by the good agreement seen between the imino ${}^{1}$H chemical shifts and those expected based on duplexes containing Hoogsteen bp's as the dominant GS conformation. The high throughput nature of the experiment and simple sample requirements enabled us to measure Hoogsteen dynamics for 37 data points corresponding to 22 distinct bp's for three different pH conditions and seven different temperatures (Table S1), the largest collection of Hoogsteen dynamics from a single study to date. We envision using the ${}^{1}$H CEST SELOPE experiments to pre-screen DNA duplexes and to perform follow-up ${}^{13}$C and ${}^{15}$N RD experiments to confirm any interesting outliers, particularly regions showing substantially elevated Hoogsteen dynamics.

An important consideration when applying ${}^{1}$H CEST to the study of chemical exchange are contributions due to ${}^{1}$H–${}^{1}$H cross-relaxation originating from cross-relaxation, which may give rise to extraneous NOE dips that complicate data analysis (Yuwen et al., 2017a; Bouvignies and Kay, 2012; Eichmuller and Skrynnikov, 2005). These contributions have been shown to be significant in proteins particularly when characterizing slow exchange ($k_{\mathrm{ex}}$ < 200 s${}^{-1}$) necessitating use of relatively long relaxation delays (Bouvignies and Kay, 2012). Consistent with prior studies of nucleic acids (Schlagnitweit et al., 2018; Steiner et al., 2016; Baronti et al., 2020) and proteins (Lundstrom and Akke, 2005). Our results indicate that NOE effects from cross-relaxation between imino and non-imino protons can be effectively suppressed for DNA and RNA duplexes in the ${}^{1}$H CEST experiments through selective excitation provided that the relaxation delays are short on the order of 100 ms (Fig. 3b). However, care should be exercised to assess imino–imino NOE effects (Fig. 3b), which may also be more substantial for certain non-canonical motifs. Data should be discarded if the ES chemical shifts match those of nearby imino protons identified using 2D [${}^{1}$H, ${}^{1}$H] NOESY experiments or if the magnitude of the dip of interest varies substantially with or without selective excitation, as this could be an indication of NOE effect. Finally, we recommend independent verification of the exchange parameters with the use of other methods such as ${}^{13}$C and ${}^{15}$N experiments for motifs exhibiting highly unusual exchange parameters or ES ${}^{1}$H chemical shifts, and this can also help to confirm Hoogsteen bp's as the ES.

Prior studies showed that Watson–Crick to Hoogsteen bp transitions exhibit large variations in the forward rate constants ($k_{1}$), while the backward rate constants ($k_{-1}$) are relatively constant across different sequence contexts, consistent with a late transitional state (Alvey et al., 2014). We observe a similar trend in which $k_{-1}$ varied < 5-fold, while $k_{1}$ varied by ∼ 50-fold (Fig. S9). The ${}^{1}$H CEST data also revealed significantly lower Hoogsteen abundance ($p_{\mathrm{ES}}$ < 0.1 %) in addition to slower exchange kinetics ($k_{\mathrm{ex}}$ ∼ 1000 s${}^{-1}$) within A-tract motifs (Nikolova et al., 2011; Alvey et al., 2014) while also reinforcing prior data (Xu et al., 2018), suggesting increased exchange kinetics near terminal ends. Collectively, these data show that the Hoogsteen population can vary by as much as ∼ 14-fold, while $k_{\mathrm{ex}}$ can vary by ∼ 20-fold only due to changes in sequence and positional context (Table S1). These strong sequence and position dependencies could play important roles in biochemical processes acting on DNA.

A recent study (Furukawa et al., 2021) reported on-resonance imino ${}^{1}$H $R_{1\rho }$ RD for a guanine residue in a DNA duplex at pH = 7.5, T = 30 ${}^{\circ }$C, and in 150 mM NaCl. Because off-resonance measurements were not performed, only $k_{\mathrm{ex}}$ ∼ 10 000 s${}^{-1}$ could be determined, while the values of Δω and $p_{\mathrm{ES}}$ were not determined. The study noted that a Hoogsteen bp as the ES was unlikely given that G–C${}^{+}$ Hoogsteen bp's are disfavored at pH = 7.5 and because the observed rate of exchange ($k_{\mathrm{ex}}$ ∼ 10 000 s${}^{-1}$) was much faster than is typically observed for Watson–Crick to Hoogsteen exchange. Instead, the data were interpreted as evidence for a base opened state. However, the observed rate of exchange $k_{\mathrm{ex}}$ ∼ 10 000 s${}^{-1}$ falls comfortably within the range of values measured here for Watson–Crick to Hoogsteen exchange using ${}^{1}$H CEST at similar pH conditions. For example, for the G10-C15 bp's in A${}_{6}$-DNA at the same temperature and pH = 6.8, $k_{\mathrm{ex}}$ for Watson–Crick to Hoogsteen exchange was ∼ 6000 s${}^{-1}$ (Fig. 4 and Table S1). Similar Watson–Crick to Hoogsteen exchange parameters ($p_{\mathrm{ES}}$ ∼ 0.05 % and $k_{\mathrm{ex}}$ ∼ 2000 s${}^{-1}$) were recently reported for this bp at 25 ${}^{\circ }$C and pH 6.8 using cytosine amino ${}^{15}$N RD (Rangadurai et al., 2019a), and the ES $\Delta \omega_{\text{C-N4}}=-9$ ppm was shown to be in excellent agreement with values expected for a G–C${}^{+}$ Hoogsteen bp. In addition, based on hydrogen exchange measurements, $p_{\mathrm{ES}}$ ∼ 0.00001 % to 0.01 % and $k_{\mathrm{ex}}$ ($k_{\mathrm{cl}}+k_{\mathrm{op}}$, $k_{\mathrm{cl}}$ and $k_{\mathrm{op}}$ are the base closing and opening rate constant, respectively) ∼ 10${}^{5}$ to 10${}^{7}$ s${}^{-1}$ for the base-opened ES, and this process should fall outside RD detection (Gueron and Leroy, 1995; Gueron et al., 1987; Leroy et al., 1988; Leijon and Graslund, 1992; Snoussi and Leroy, 2001). Therefore, the ES detected by Furukawa et al. (2021) is more likely a Hoogsteen bp.

In conclusion, by obviating the need for isotopic enrichment, the ${}^{1}$H CEST experiment expands the scope of characterizing Watson–Crick to Hoogsteen exchange in nucleic acids by NMR. We are presently applying the experiment to map the sequence dependence of Hoogsteen breathing dynamics and systematically, how it varies with pH, salt and crowding, and following the introduction of lesions, mismatches and molecules that bind to the DNA.

## 4 Methods

### 4.1 Sample preparation

*Unlabeled DNA and RNA oligonucleotides*. Unmodified DNA oligonucleotides were purchased from Integrated DNA Technologies with standard desalting purification. RNA oligonucleotides were synthesized using a MerMade 6 Oligo Synthesizer employing 2${}^{\prime }$-tBDSilyl protected phosphoramidites (n-acetyl protected rC, rA and rG, and rU phosphoramidites were purchased from ChemGenes) and 1 µmol standard synthesis columns (1000 Å) (BioAutomation). RNA oligonucleotides were synthesized with the final 5${}^{\prime }$-protecting group, 4,4${}^{\prime }$-dimethoxytrityl (DMT) retained. RNA oligonucleotides were cleaved from columns using 1 mL AMA (1 : 1 ratio of 30 % ammonium hydroxide and 30 % methylamine) and incubated at room temperature for 2 h. The sample was then air-dried and dissolved in 115 µL DMSO, 60 µL TEA, and 75 µL TEA.3HF, and then incubated at T = 65 ${}^{\circ }$C for 2.5 h to remove 2${}^{\prime }$-O protecting groups. The Glen-Pak RNA cartridges (Glen Research Corporation) were then used to purify the samples followed by ethanol precipitation.

*Labeled DNA oligonucleotides*. The uniformly ${}^{13}$C, ${}^{15}$N-labeled A${}_{6}$-DNA sample was prepared using chemically synthesized DNA (purchased from IDT), Klenow fragment DNA polymerase (New England Biolab) and ${}^{13}$C / ${}^{15}$N isotopically labeled dNTPs (Silantes) using the Zimmer and Crothers method (Zimmer and Crothers, 1995). The oligonucleotide was purified using 20 % 29 : 1 polyacrylamide denaturing gel with 8 M urea, 20 mM Tris borate and 1 mM EDTA, and then using electro-elution (Whatman, GE Healthcare) in 40 mM Tris acetate and 1 mM EDTA, followed by ethanol precipitation.

*Sample annealing and buffer exchange*. DNA/RNA oligonucleotides were re-suspended in water (200–500 µM). To prepare duplex samples, equimolar amounts of the constituent single-stranded DNA/RNA samples were mixed and then heated at T = 95 ${}^{\circ }$C for ∼ 5 min followed by cooling at room temperature for ∼ 1 h. All samples were exchanged three times into the desired buffer using centrifugal concentrators (4 mL, Millipore Sigma). A total of 10 % D${}_{2}$O (Millipore Sigma) was added to the samples prior to the NMR measurements.

*Sample concentrations and buffer conditions*. Unless mentioned otherwise, the NMR buffer contains 25 mM sodium chloride, 15 mM sodium phosphate, 0.1 mM EDTA and 10 % D${}_{2}$O. Sample concentrations and buffer pH are as follows: A${}_{6}$-DNA, 1.0 mM, pH 6.8; A${}_{2}$-DNA, 1.0 mM, pH 5.4; A${}_{5}$-DNA, 0.2 mM, pH 5.2; A${}_{6}$-RNA, 0.5 mM, pH 6.8. Concentration was estimated by measuring the absorbance of the sample at 260 nm and using extinction coefficients from the ADT Biol Oligo calculator (https://www.atdbio.com/tools/oligo-calculator, last access: 12 September 2021).

### 4.2 NMR spectroscopy

All NMR experiments were performed on a 600 Bruker Avance 3 spectrometer equipped with a triple-resonance HCN cryogenic probe. The NMR data were processed and analyzed with NMRPipe (Delaglio et al., 1995) and SPARKY (Thomas D. Goddard and Donald G. Kneller​​​​​​​, SPARKY 3, University of California, San Francisco).

*Resonance assignments*. Imino resonances were assigned using a combination of 2D [${}^{1}$H, ${}^{1}$H] NOESY and [${}^{15}$N,${}^{1}$H] SOFAST-HMQC (Sathyamoorthy et al., 2014) experiments. Assignments for A${}_{6}$-DNA, A${}_{2}$-DNA and A${}_{6}$-RNA were reported previously (Sathyamoorthy et al., 2017; Zhou et al., 2016; Nikolova et al., 2011). The [${}^{1}$H, ${}^{1}$H] NOESY spectrum for A${}_{5}$-DNA is shown in Fig. S1.

${}^{1}$*H CEST*. The pulse sequence is shown in Fig. 1b and was adapted from Schlagnitweit et al. (2018). The g1 gradient (Fig. 1b) destroys transverse ${}^{1}$H magnetization prior to excitation of imino resonances. This helps to avoid any accidental offset dependence of the starting ${}^{1}$H magnetization. Relaxation delays $T_{\mathrm{EX}}=100$ ms were used for all ${}^{1}$H CEST measurements at low temperatures (5–30 ${}^{\circ }$C), while a shorter $T_{\mathrm{EX}}=80$ ms was used for high (45 ${}^{\circ }$C) temperature measurements. A longer $T_{\mathrm{EX}}=400$ ms was used to illustrate artifacts arising due to NOE dips (Fig. 3b). RF power and offset combinations used in the CEST measurements are given in Table S2. Calibration of RF field powers for the ${}^{1}$H CEST measurements was performed as described previously (Rangadurai et al., 2019b) using the same pulse sequence. Field inhomogeneity was also measured (Fig. S10) using the same sequence and the procedure as described previously (Guenneugues et al., 1999). ${}^{1}$H inhomogeneity was measured by performing on-resonance ${}^{1}$H CEST experiments on G2-H1 of A${}_{6}$-DNA, chosen as it does not experience conformational exchange. The longest relaxation delays used for the measurements were 10, 2, 1, 0.4, 0.1 and 0.04 s for RF fields 10, 50, 100, 200, 1000 and 4000 Hz, respectively. The resulting nutation curve was Fourier transformed and was fit to a Gaussian function (blue lines in Fig. S10) to extract the full width at half maximum, which was used for defining the inhomogeneity as described previously (Guenneugues et al., 1999). The selective pulse was set to be off (Fig. 3b) by replacing pulse a (Fig. 1b) with a non-selective ${}^{1}$H hard 90${}^{\circ }$ pulse. A total of 16 scans were used for A${}_{6}$-DNA (1.0 mM) at 5, 10, 20, 25, 30 ${}^{\circ }$C, and A${}_{2}$-DNA (1.0 mM) at 25 ${}^{\circ }$C. A total of 32 scans were used for A${}_{6}$-RNA (0.5 mM) at 25 ${}^{\circ }$C. A total of 64 scans were used for A${}_{5}$-DNA (0.2 mM) at 25 ${}^{\circ }$C and for A${}_{6}$-DNA (1.0 mM) at 45 ${}^{\circ }$C.

*Fitting of* ${}^{1}$*H CEST data*. When performing two-state CEST fitting with and without exchange, we restricted the offset to -6 to 6 ppm for the ${}^{1}$H CEST experiment with relaxation delay ≤ 100 ms and to -3 to 3 ppm for experiments with relaxation delay = 400 ms, to obviate any potential effects from ${}^{1}$H–${}^{1}$H cross-relaxation artifacts (Fig. 3b). Peak intensities of all imino protons in the 1D spectra as a function of RF power and offset frequency were extracted using NMRPipe (Delaglio et al., 1995). The peak intensity at a given RF power and offset is normalized by the average peak intensity over the triplicate CEST measurements with zero relaxation delay under the same RF power. The uncertainty in the measured peak intensity at each offset frequency and RF power combination was assumed to be equal to the standard deviation of the peak intensities for triplicate CEST experiments with zero relaxation delay under the same RF power (Zhao et al., 2014; Shi et al., 2019). CEST profiles were generated by plotting the normalized intensity as a function of offset $\Omega =\omega_{\mathrm{RF}}-\omega_{\mathrm{obs}}$, where $\omega_{\mathrm{obs}}$ is the Larmor frequency of the observed resonance and $\omega_{\mathrm{RF}}$ is the angular frequency of the applied RF field. RF field inhomogeneity (Fig. S10) was taken into account during CEST fitting as described previously (Rangadurai et al., 2020a). The normalized CEST profiles were then fit via numerical integration of the Bloch–McConnell (B–M) equations as described previously (Rangadurai et al., 2020a). Fitting of CEST profiles without exchange (Figs. 4, S2–S4) was performed by setting $p_{\mathrm{ES}}=k_{\mathrm{ex}}=\Delta \omega =0$. Errors in exchange parameters were set to be equal to the fitting errors which were obtained as the square root of the diagonal elements of the covariance matrix. Reduced chi-square ($r\chi^{2}$) was calculated to assess the goodness of fit (Rangadurai et al., 2019b). Note that the variations in $r\chi^{2}$ values for different ${}^{1}$H CEST profiles in Fig. 4 and Fig. S2–4 are most likely due to differences in the quality of the NMR data and poor estimation of the real experimental uncertainty. The residual sum of squares (RSS) was computed as follows:

1 $$\mathrm{RSS}=\sum_{i=1}^{n}{\left(I_{i}^{\mathrm{fit}}-I_{i}^{\mathrm{exp}}\right)}^{2},$$

where $I_{i}^{\mathrm{fit}}$ and $I_{i}^{\mathrm{exp}}$ are the ith fit and experimentally measured intensity in the CEST profile respectively, and the summation is over all RF power and offset combinations (N).

Model selection for fits with and without exchange (Figs. 4, S2–S4) was performed by computing AIC and BIC weights as follows (Burnham and Anderson, 2004):

2AIC=Nln⁡RSSN+2K,whenNK≥40Nln⁡RSSN+2K+2KK+1N-K-1,whenNK<403wAIC=e-0.5ΔAIC1+e-0.5ΔAIC4BIC=Nln⁡RSSN+Kln⁡N5wBIC=e-0.5ΔBIC1+e-0.5ΔBIC,

where K is the number of floating parameters when fitting, and ΔAIC/ΔBIC are the differences between two AIC values (fitting without and with exchange). The AIC (wAIC${}_{+\mathrm{ex}}$) and BIC (wBIC${}_{+\mathrm{ex}}$) weights for fits with exchange are reported in Figs. 4 and S2–S4. The improvement in the fit was considered statistically significant if both wAIC${}_{+\mathrm{ex}}$ and wBIC${}_{+\mathrm{ex}}$ values are > 0.995, and $r\chi^{2}$ is reduced with the inclusion of exchange. For some resonances, the improvement in the fit with exchange is statistically significant, but the resulting exchange parameters are not reliable and have large errors (see Figs. S2, S3). For T4 in A${}_{5}$-DNA, $p_{\mathrm{ES}}=0.2$ ± 0.1 % measured using ${}^{1}$H CEST was ∼ 10-fold smaller than $p_{\mathrm{ES}}=2.7$ ± 1.5 % measured previously using ${}^{15}$N RD (Alvey et al., 2014), whereas $k_{\mathrm{ex}}$ (∼ 3000 s${}^{-1}$) was in good agreement. However, simulations show that due to the small Δω for ${}^{15}$N (∼ 1 ppm) and fast exchange kinetics $k_{\mathrm{ex}}$ (∼ 3000 s${}^{-1}$) the $p_{\mathrm{ES}}$ and Δω are not well-determined by the ${}^{15}$N RD data (Fig. S6c). For this reason, this data point was excluded for ${}^{1}$H CEST and ${}^{13}$C / ${}^{15}$N RD comparison (Fig. 5a).

*Off-resonance* ${}^{13}CR_{1\rho }$ *relaxation dispersion*. ${}^{13}$C $R_{1\rho }$ experiments were performed using 1D $R_{1\rho }$ schemes as described previously (Nikolova et al., 2012a, 2011; Hansen et al., 2009). The spin-lock powers and offsets are listed in Table S3. The spin-lock was applied for a maximal duration < 60 ms to achieve ∼ 70 % loss of peak intensity at the end of relaxation delay. Off-resonance $R_{1\rho }$ profiles (Fig. S8) were generated by plotting $(R_{2}+R_{\mathrm{ex}})=(R_{1\rho }-R_{1}{\mathrm{cos}}^{2}\theta )/{\mathrm{sin}}^{2}\theta$, where θ is the angle between the effective field of the observed resonance and the z axis, as a function of $\Omega_{\mathrm{eff}}/2\pi$, where $\Omega_{\mathrm{eff}}=\omega_{\mathrm{obs}}-\omega_{\mathrm{RF}}$, where $\omega_{\mathrm{obs}}$ is the Larmor frequency of the spin and $\omega_{\mathrm{RF}}$ is the carrier frequency of the applied spin-lock.

*Fitting of* ${}^{13}CR_{1\rho }$ *data*. One-dimensional peak intensities were measured using NMRPipe (Delaglio et al., 1995). $R_{1\rho }$ values for a given spin-lock power and offset were calculated by fitting the intensities as a function of delay time to a mono-exponential decay (Kimsey et al., 2015). A Monte Carlo approach was used to calculate the uncertainties of $R_{1\rho }$ (Bothe et al., 2014). Alignment of initial magnetization during the Bloch–McConnell fitting was performed based on the $k_{\mathrm{ex}}/\Delta \omega$ value (Rangadurai et al., 2019b). Chemical exchange parameters were obtained by fitting experimental $R_{1\rho }$ values to numerical solutions of a two-state Bloch–McConnell (B–M) equations (Mcconnell, 1958). A Monte Carlo approach was used to calculate the errors of exchange parameters (Bothe et al., 2014) . Reduced chi-square ($r\chi^{2}$) was calculated to assess the goodness of fit (Rangadurai et al., 2019b).

### 4.3 Thermodynamic Analysis

The observed temperature dependence of $k_{1}$, $k_{-1}$ for the Watson–Crick to Hoogsteen exchange measuring using ${}^{1}$H CEST was fit to a modified van 't Hoff equation that accounts for statistical compensation effects and assumes a smooth energy surface as described previously (Nikolova et al., 2011; Coman and Russu, 2005):

6 $$\begin{array}{rl} \mathrm{ln}\left(\frac{k_{i}\left(T\right)}{T}\right) & =\mathrm{ln}\left(\frac{k_{B}\kappa }{h}\right)-\frac{\Delta G_{i}^{\circ T}\left(T_{\mathrm{hm}}\right)}{RT_{\mathrm{hm}}}\\ & -\frac{\Delta H_{i}^{\circ T}}{R}\left(\frac{1}{T}-\frac{1}{T_{\mathrm{hm}}}\right). \end{array}$$

$k_{i}$ (i = 1, -1) is the forward and backward rate constants, and $\Delta G_{i}^{\circ T}(T)$ and $\Delta H_{i}^{\circ T}$ are the free energy (at temperature T, in kelvin) and enthalpy of activation (i = 1) or deactivation (i = -1) respectively. R is the universal gas constant (kcal mol${}^{-1}$ K${}^{-1}$) and $T_{\mathrm{hm}}$ is the harmonic mean of the experimental temperatures ($T_{i}$ in K) computed as $T_{\mathrm{hm}}=n/\sum_{i=1}^{n}(1/T_{i})$, $k_{B}$ is the Boltzmann's constant (J K${}^{-1}$), κ is the transmission coefficient (assumed to be unity), and h is the Planck constant (J s).

The goodness-of-fit indicator $R^{2}$ (coefficient of determination) (Fig. S6) between the measured and fitted rate constants was calculated as follows: $R^{2}=1-\frac{{\mathrm{SS}}_{\mathrm{res}}}{{\mathrm{SS}}_{\mathrm{total}}}$, ${\mathrm{SS}}_{\mathrm{res}}=\sum {\left(k_{i,\mathrm{fit}}-k_{i,\mathrm{exp}}\right)}^{2}$, ${\mathrm{SS}}_{\mathrm{total}}=\sum {\left(k_{i,\mathrm{exp}}-\bar{k_{i,\mathrm{exp}}}\right)}^{2}$. $k_{i,\mathrm{fit}}$ and $k_{i,\mathrm{exp}}$ (i = 1, -1) are fitted and experimentally measured rate constants. $\bar{k_{i,\mathrm{exp}}}$ is the mean of all $k_{i,\mathrm{exp}}$. Errors of fitting for $\Delta G_{i}^{\circ T}$ and $\Delta H_{i}^{T}$ were calculated as the square root of the diagonal elements of the covariance matrix. $T\Delta S_{i}^{T}$ is calculated as $\Delta H_{i}^{T}-\Delta G_{i}^{\circ T}$.

## Supplement

10.5194/mr-2-715-2021-supplement The supplement related to this article is available online at: https://doi.org/10.5194/mr-2-715-2021-supplement.

## Data Availability

The data that support this study are contained in the published article (Tables S1–S3) or are available from the corresponding author on reasonable request. The Python scripts for H CEST data fitting are available at (last access: 12 September 2021) (DOI: , Liu et al., 2021b).
