# Supplementary material for: Rapid assessment of Watson–Crick to Hoogsteen exchange in unlabeled DNA duplexes using high-power SELOPE imino 1H CEST
Source: Magn Reson (Gott). 2021 Sep 14;2(2):715–31. doi: 10.5194/mr-2-715-2021 (PMC10539785; doi:10.5194/mr-2-715-2021)
Supplement: The supplement related to this article is available online at: https://doi.org/10.5194/mr-2-715-2021-supplement. [file mr-2-715-supplement.pdf]

Supplement of Magn. Reson., 2, 715–731, 2021
https://doi.org/10.5194/mr-2-715-2021-supplement
© Author(s) 2021. CC BY 4.0 License.

Supplement of

Rapid assessment of Watson–Crick to Hoogsteen exchange in unlabeled DNA duplexes using high-power SELOPE imino ^{1}H CEST

Bei Liu et al.

Correspondence to: Hashim M. Al-Hashimi (hashim.al.hashimi@duke.edu)

The copyright of individual parts of the supplement might differ from the article licence.

Supplementary figures

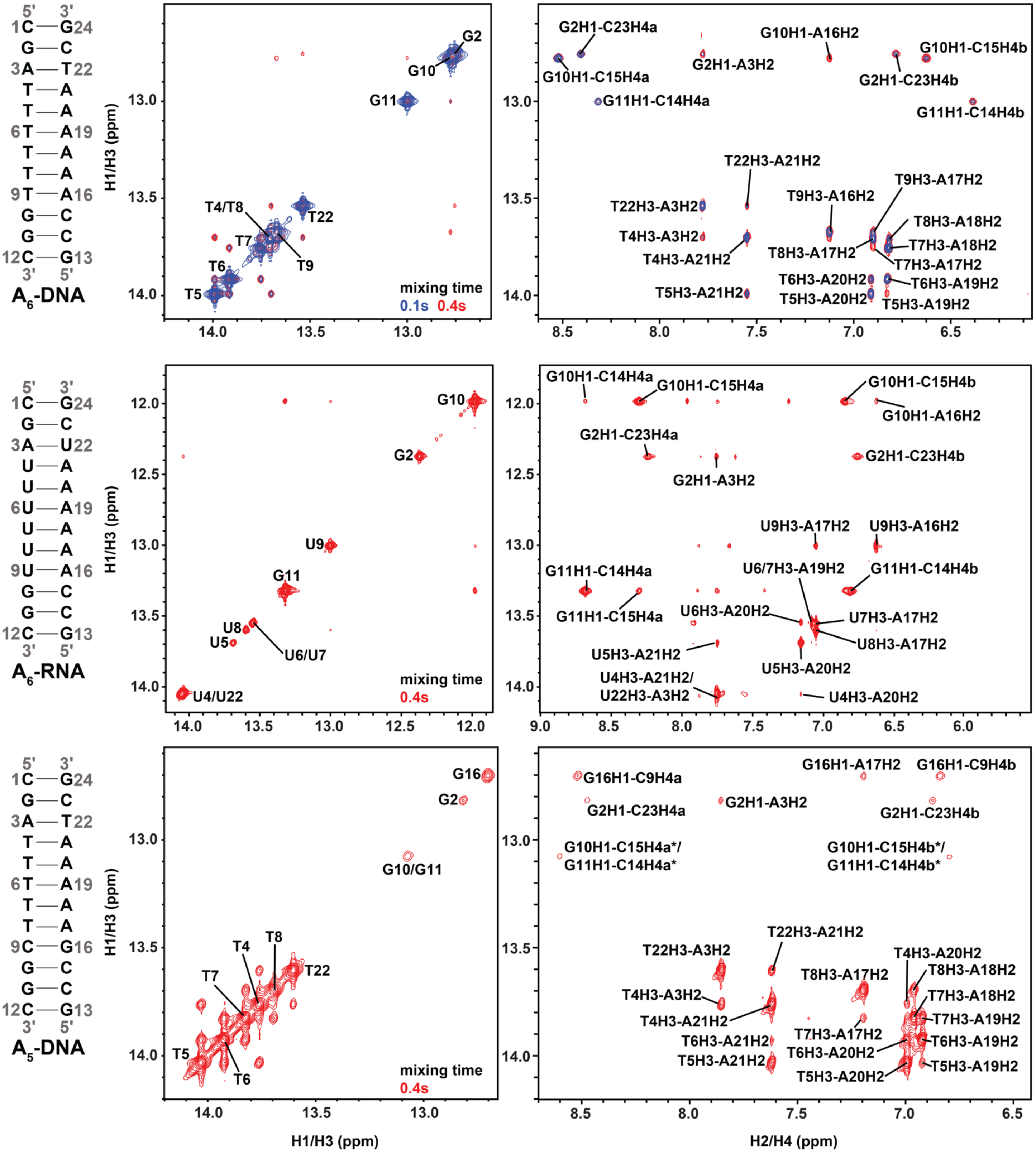

Fig. S1. 2D [^{1}H, ^{1}H] NOESY spectra for duplexes used in this study. Shown are imino-imino and imino-amino/aromatic regions of 2D [^{1}H, ^{1}H] NOESY spectra of A_6-DNA, A_6-RNA and A_5-DNA. Mixing times used are indicated in inset.

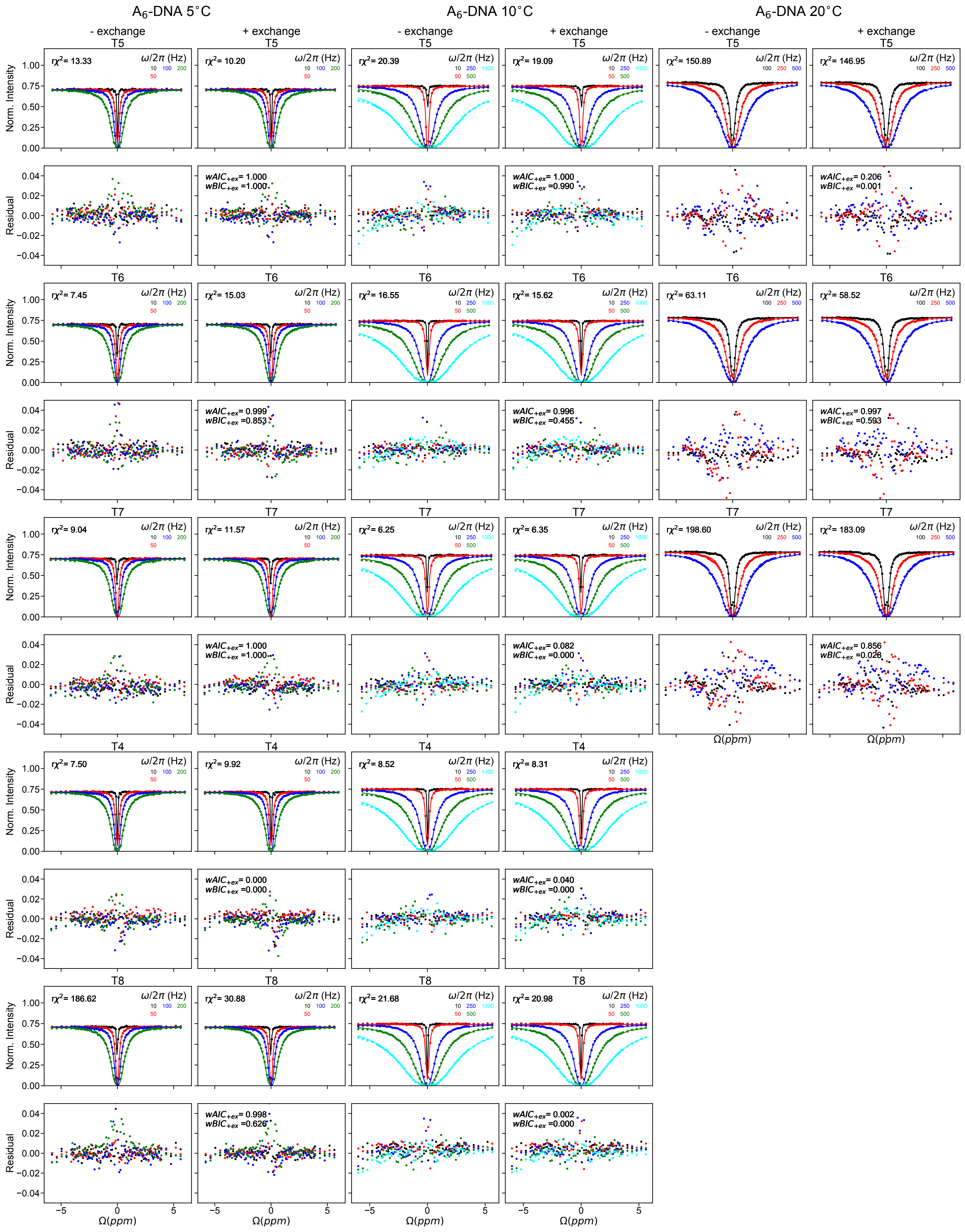

A6-DNA 5°C
A6-DNA 10°C
A6-DNA 20°C
- exchange
+ exchange
T5
T6
T7
T4
T8
Norm. Intensity
Residual
Ω(ppm)
ω/2π (Hz)
10 50 100 200
10 50 250 500 1000
100 250 500
rχ2= 13.33
rχ2= 10.20
wAIC+ex= 1.000
wBIC+ex =1.000
rχ2= 20.39
rχ2= 19.09
wAIC+ex= 1.000
wBIC+ex =0.990
rχ2= 150.89
rχ2= 146.95
wAIC+ex= 0.206
wBIC+ex =0.001
rχ2= 7.45
rχ2= 15.03
wAIC+ex= 0.999
wBIC+ex =0.853
rχ2= 16.55
rχ2= 15.62
wAIC+ex= 0.996
wBIC+ex =0.455
rχ2= 63.11
rχ2= 58.52
wAIC+ex= 0.997
wBIC+ex =0.593
rχ2= 9.04
rχ2= 11.57
wAIC+ex= 1.000
wBIC+ex =1.000
rχ2= 6.25
rχ2= 6.35
wAIC+ex= 0.082
wBIC+ex =0.000
rχ2= 198.60
rχ2= 183.09
wAIC+ex= 0.856
wBIC+ex =0.028
rχ2= 7.50
rχ2= 9.92
wAIC+ex= 0.000
wBIC+ex =0.000
rχ2= 8.52
rχ2= 8.31
wAIC+ex= 0.040
wBIC+ex =0.000
rχ2= 186.62
rχ2= 30.88
wAIC+ex= 0.998
wBIC+ex =0.626
rχ2= 21.68
rχ2= 20.98
wAIC+ex= 0.002
wBIC+ex =0.000

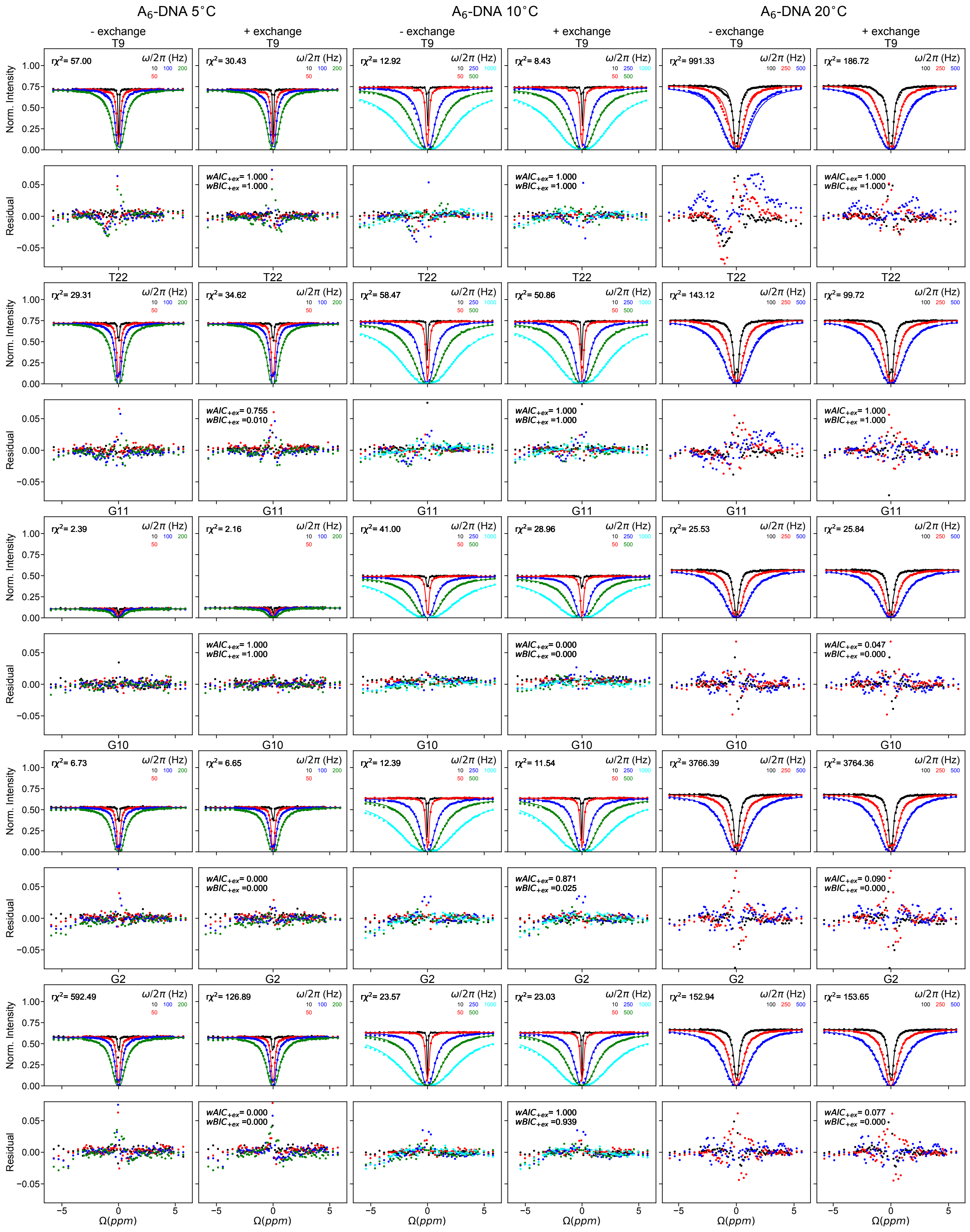

A6-DNA 5°C
A6-DNA 10°C
A6-DNA 20°C
- exchange
+ exchange
T9
T22
G11
G10
G2
Norm. Intensity
Residual
Ω(ppm)
ω/2π (Hz)
rχ2= 57.00
rχ2= 30.43
rχ2= 12.92
rχ2= 8.43
rχ2= 991.33
rχ2= 186.72
wAIC+ex= 1.000
wBIC+ex =1.000
rχ2= 29.31
rχ2= 34.62
rχ2= 58.47
rχ2= 50.86
rχ2= 143.12
rχ2= 99.72
wAIC+ex= 0.755
wBIC+ex =0.010
rχ2= 2.39
rχ2= 2.16
rχ2= 41.00
rχ2= 28.96
rχ2= 25.53
rχ2= 25.84
wAIC+ex= 0.000
wBIC+ex =0.000
wAIC+ex= 0.047
wBIC+ex =0.000
rχ2= 6.73
rχ2= 6.65
rχ2= 12.39
rχ2= 11.54
rχ2= 3766.39
rχ2= 3764.36
wAIC+ex= 0.871
wBIC+ex =0.025
wAIC+ex= 0.090
wBIC+ex =0.000
rχ2= 592.49
rχ2= 126.89
rχ2= 23.57
rχ2= 23.03
rχ2= 152.94
rχ2= 153.65
wAIC+ex= 1.000
wBIC+ex =0.939
wAIC+ex= 0.077
wBIC+ex =0.000

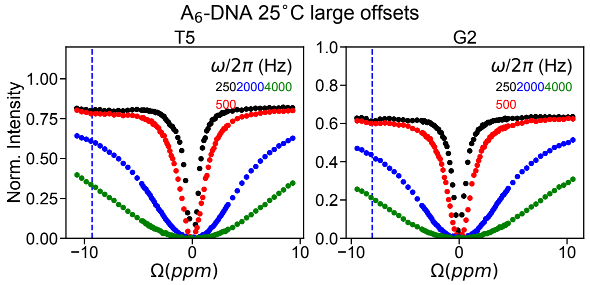
A6-DNA 25°C large offsets
T5
G2
ω/2π (Hz)
250 2000 4000
500
Norm. Intensity
Ω(ppm)

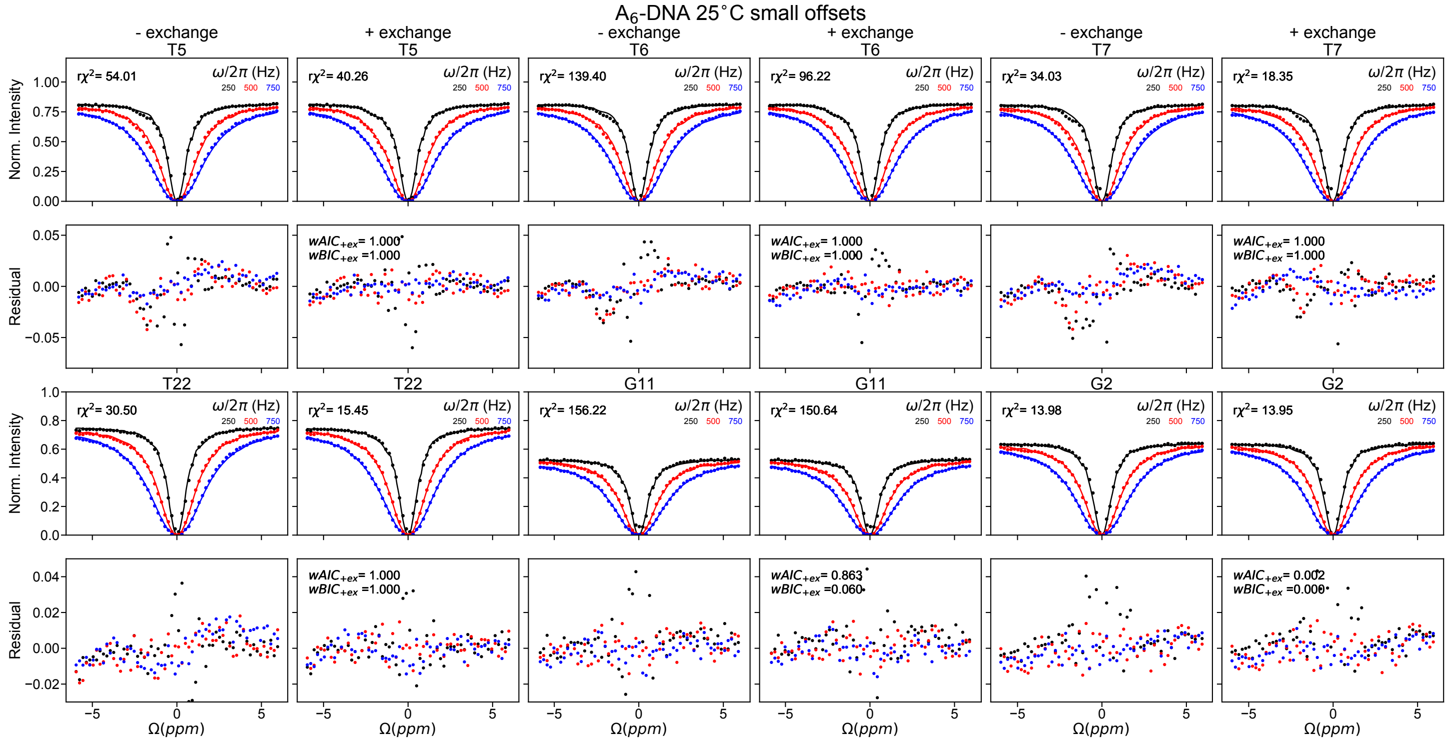
A6-DNA 25°C small offsets
- exchange
+ exchange
T5
T6
T7
T22
G11
G2
rχ2= 54.01
rχ2= 40.26
rχ2= 139.40
rχ2= 96.22
rχ2= 34.03
rχ2= 18.35
rχ2= 30.50
rχ2= 15.45
rχ2= 156.22
rχ2= 150.64
rχ2= 13.98
rχ2= 13.95
ω/2π (Hz)
250 500 750
wAIC+ex= 1.000
wBIC+ex =1.000
wAIC+ex= 0.863
wBIC+ex =0.060
wAIC+ex= 0.002
wBIC+ex =0.000
Norm. Intensity
Residual
Ω(ppm)

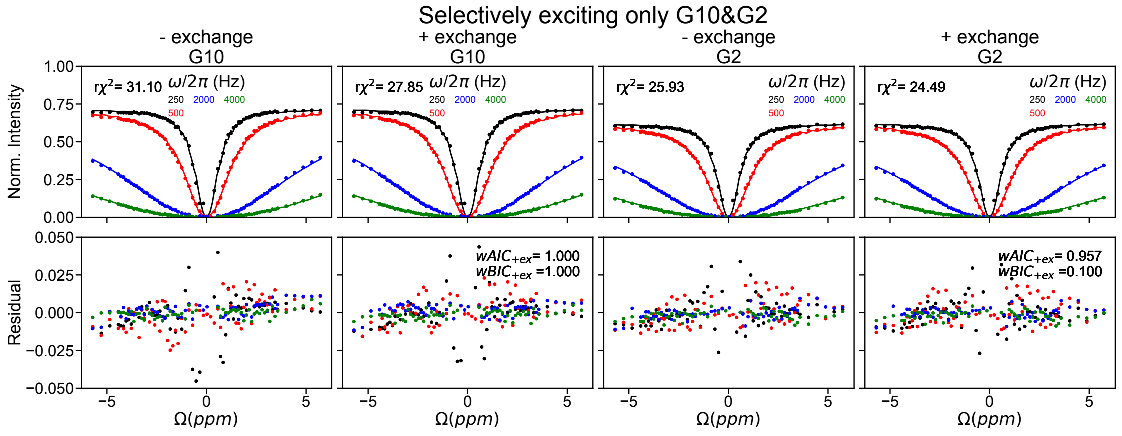
Selectively exciting only G10&G2
- exchange
+ exchange
G10
G2
rχ2= 31.10
rχ2= 27.85
rχ2= 25.93
rχ2= 24.49
ω/2π (Hz)
250 2000 4000
500
wAIC+ex= 1.000
wBIC+ex =1.000
wAIC+ex= 0.957
wBIC+ex =0.100
Norm. Intensity
Residual
Ω(ppm)

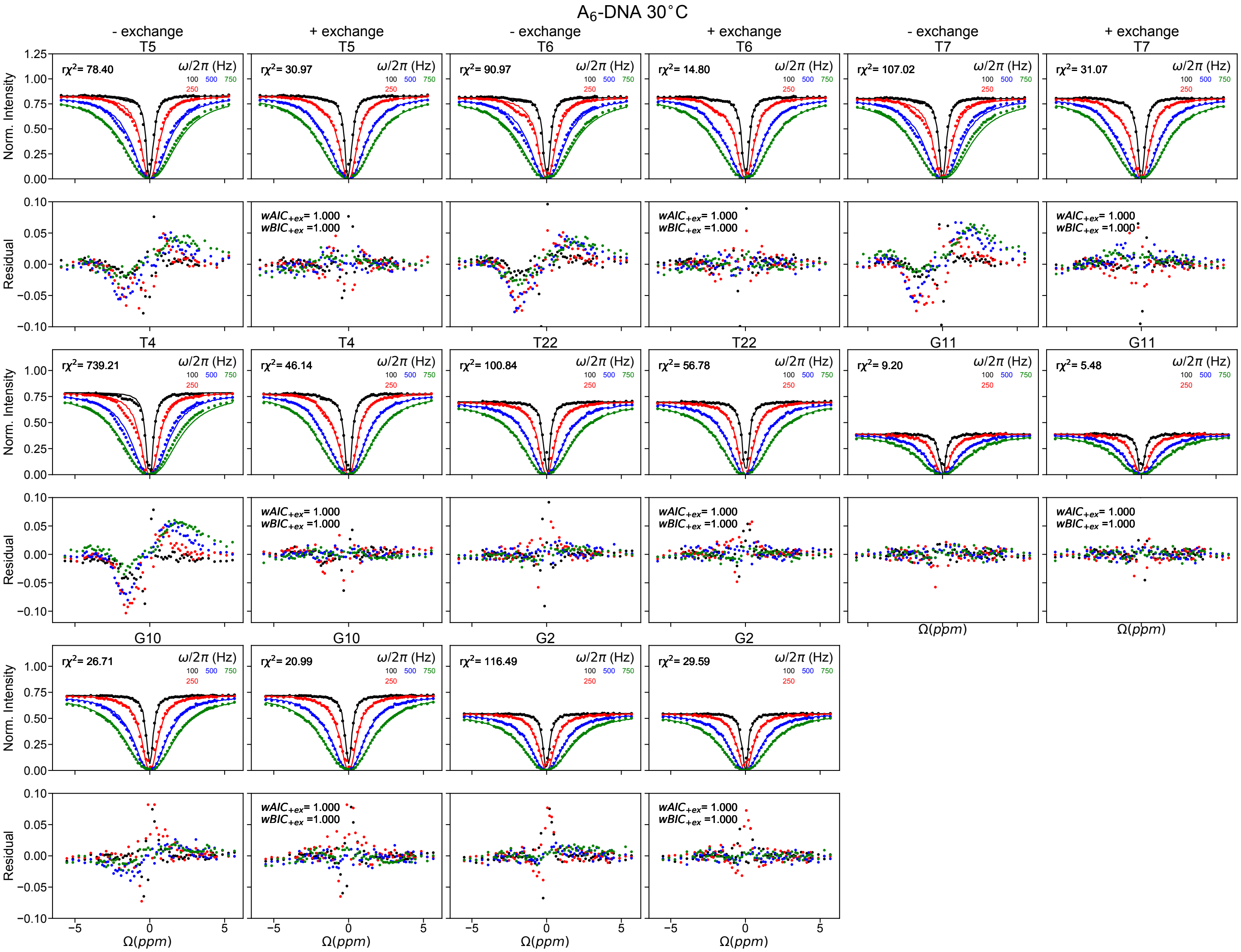
A$_6$-DNA 30°C
- exchange T5
+ exchange T5
- exchange T6
+ exchange T6
- exchange T7
+ exchange T7
rχ^2= 78.40
rχ^2= 30.97
rχ^2= 90.97
rχ^2= 14.80
rχ^2= 107.02
rχ^2= 31.07
ω/2π (Hz)
100 250 500 750
Norm. Intensity
Residual
wAIC$_{+ex}$= 1.000
wBIC$_{+ex}$ =1.000
T4
T22
G11
rχ^2= 739.21
rχ^2= 46.14
rχ^2= 100.84
rχ^2= 56.78
rχ^2= 9.20
rχ^2= 5.48
G10
G2
rχ^2= 26.71
rχ^2= 20.99
rχ^2= 116.49
rχ^2= 29.59
Ω(ppm)

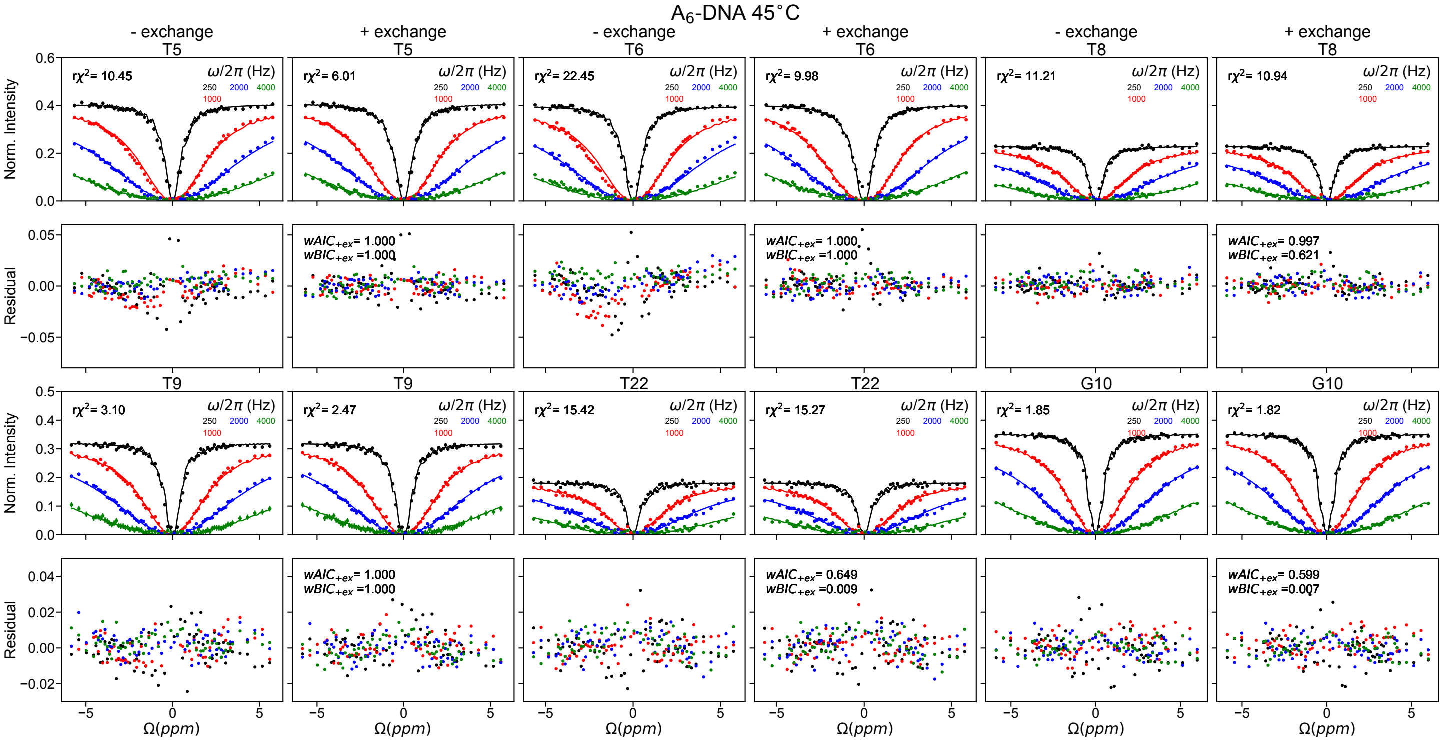
A$_6$-DNA 45°C
- exchange T5
+ exchange T5
- exchange T6
+ exchange T6
- exchange T8
+ exchange T8
rχ^2= 10.45
rχ^2= 6.01
rχ^2= 22.45
rχ^2= 9.98
rχ^2= 11.21
rχ^2= 10.94
ω/2π (Hz)
250 1000 2000 4000
Norm. Intensity
Residual
wAIC$_{+ex}$= 1.000
wBIC$_{+ex}$ =1.000
wAIC$_{+ex}$= 0.997
wBIC$_{+ex}$ =0.621
T9
T22
G10
rχ^2= 3.10
rχ^2= 2.47
rχ^2= 15.42
rχ^2= 15.27
rχ^2= 1.85
rχ^2= 1.82
wAIC$_{+ex}$= 0.649
wBIC$_{+ex}$ =0.009
wAIC$_{+ex}$= 0.599
wBIC$_{+ex}$ =0.007
Ω(ppm)

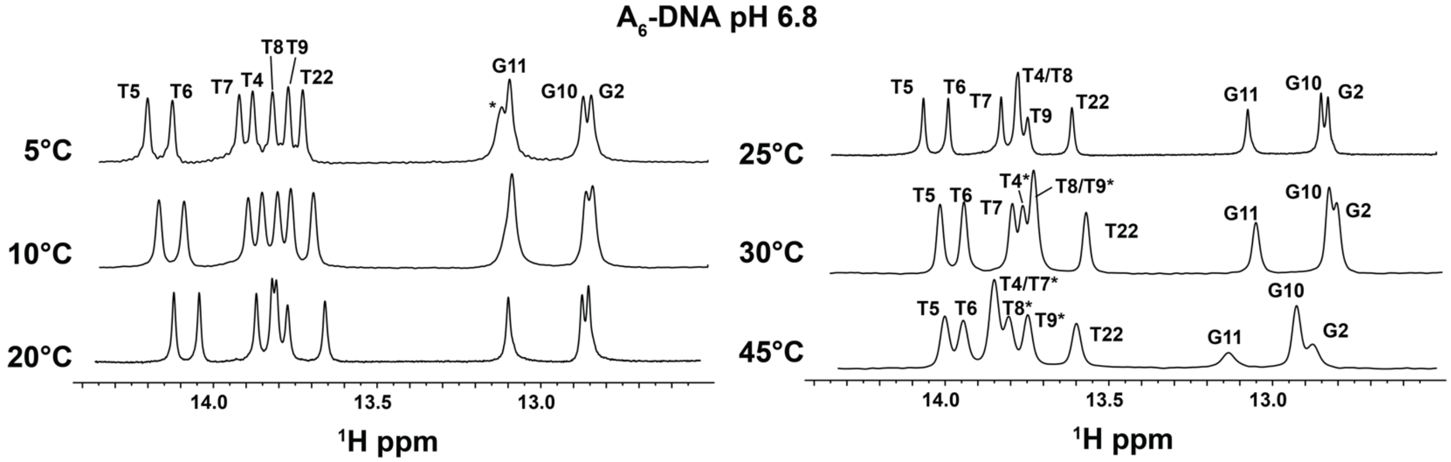

Fig. S2. ^{1}H CEST profiles and 1D imino spectra measured for A_6-DNA as a function of temperature. RF field powers used for ^{1}H CEST are color-coded. Shown are fits (solid lines) of the data (points) to the Bloch-McConnell equations with and without (k_{ex} = $\Delta\omega$ = p_{ES} = 0) 2-state chemical exchange. Shown below the CEST profiles are residual plots (normalized intensity - fitted normalized intensity). Reduced chi-square ($r\chi^2$), Akaike's (w*AIC*) and Bayesian (w*BIC*) information criterion weights were used to select the model with (+ex) or without (-ex) exchange as described in the main text. Note for the following residues, although the AIC/BIC weights > 0.995 and $r\chi^2$ is reduced with the inclusion of exchange, the exchange parameters are not reliable/have large errors: G11 at 5°C (p_{ES} = 0.016±3.62, $\Delta\omega$ = -0.06±14.3 ppm, k_{ex} = 29962.9±5883345.6 s^{-1}) and G11 at 30°C (p_{ES} = 0.049±1.36, $\Delta\omega$ = -0.24±6.76 ppm, k_{ex} = 49978.8±625763.2 s^{-1}). For 25°C data, representative profiles (G2 and T5) with large offsets are also shown. The dashed blue lines indicate the water chemical shift. Also shown are the 1D ^{1}H spectra of the imino region for A_6-DNA at different temperatures. G11 and G2 imino peaks are too weak to measure ^{1}H CEST at 45°C. Error bars for ^{1}H CEST profiles (typically smaller than data points) were obtained using the standard deviation of 3 measurements of peak intensity with zero relaxation delay as described in Methods. ^{1}H CEST data for T4, T8 at 20°C and 25°C, T8, T9 at 30°C, and T4, T7 at 45°C are not shown due to spectra overlap. ^{1}H CEST data for T9 and G10 at 25°C is shown in Fig. 4.

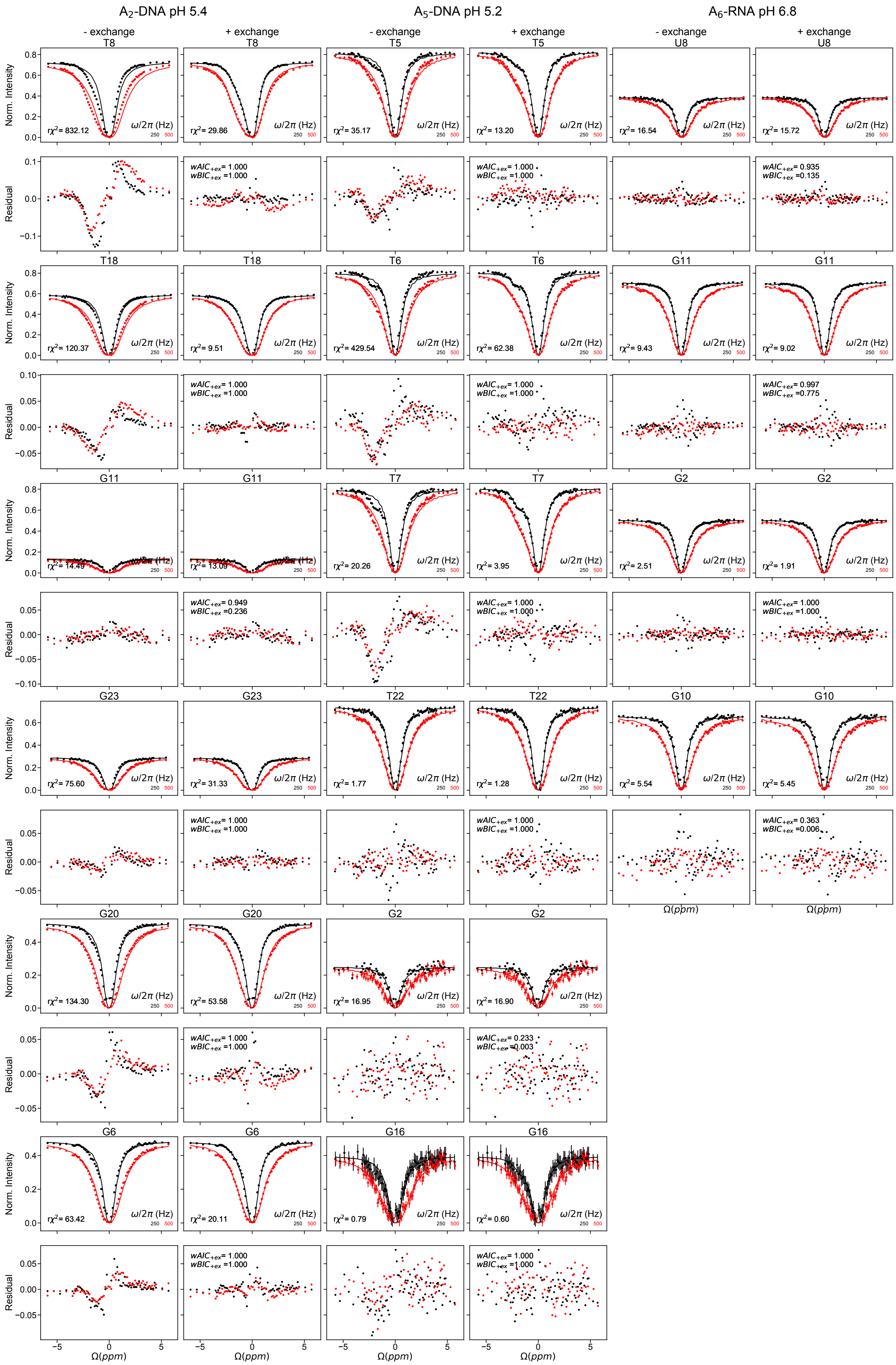

A2-DNA pH 5.4
A5-DNA pH 5.2
A6-RNA pH 6.8
- exchange
+ exchange
Norm. Intensity
Residual
Ω(ppm)
ω/2π (Hz)

Fig. S3. [1]H CEST profiles measured for A_2-DNA at 25°C, A_5-DNA at 26°C and A_6-RNA at 25°C. RF field powers used for ^{1}H CEST are color-coded. Shown are 2-state fits of the data to the Bloch-McConnell equations with and without (k_{ex} = $\Delta\omega$ = p_{ES} = 0) 2-state chemical exchange. Shown below the CEST profiles are residual plots (normalized intensity - fitted normalized intensity). Reduced chi-square ($r\chi^2$), Akaike's (w*AIC*) and Bayesian (w*BIC*) information criterion weights were used to select the model with (+ex) or without (-ex) exchange as described in the main text. Note that although G2 in A_6-RNA has AIC/BIC weights > 0.995 and $r\chi^2$ is reduced with the inclusion of exchange, the exchange parameters are not reliable (p_{ES} = 40±85%, $\Delta\omega$ = -0.03±0.05 ppm, k_{ex} = 164±553 s^{-1}). Error bars for ^{1}H CEST profiles (typically smaller than data points) were obtained using the standard deviation of 3 measurements of peak intensity with zero relaxation delay as described in Methods.

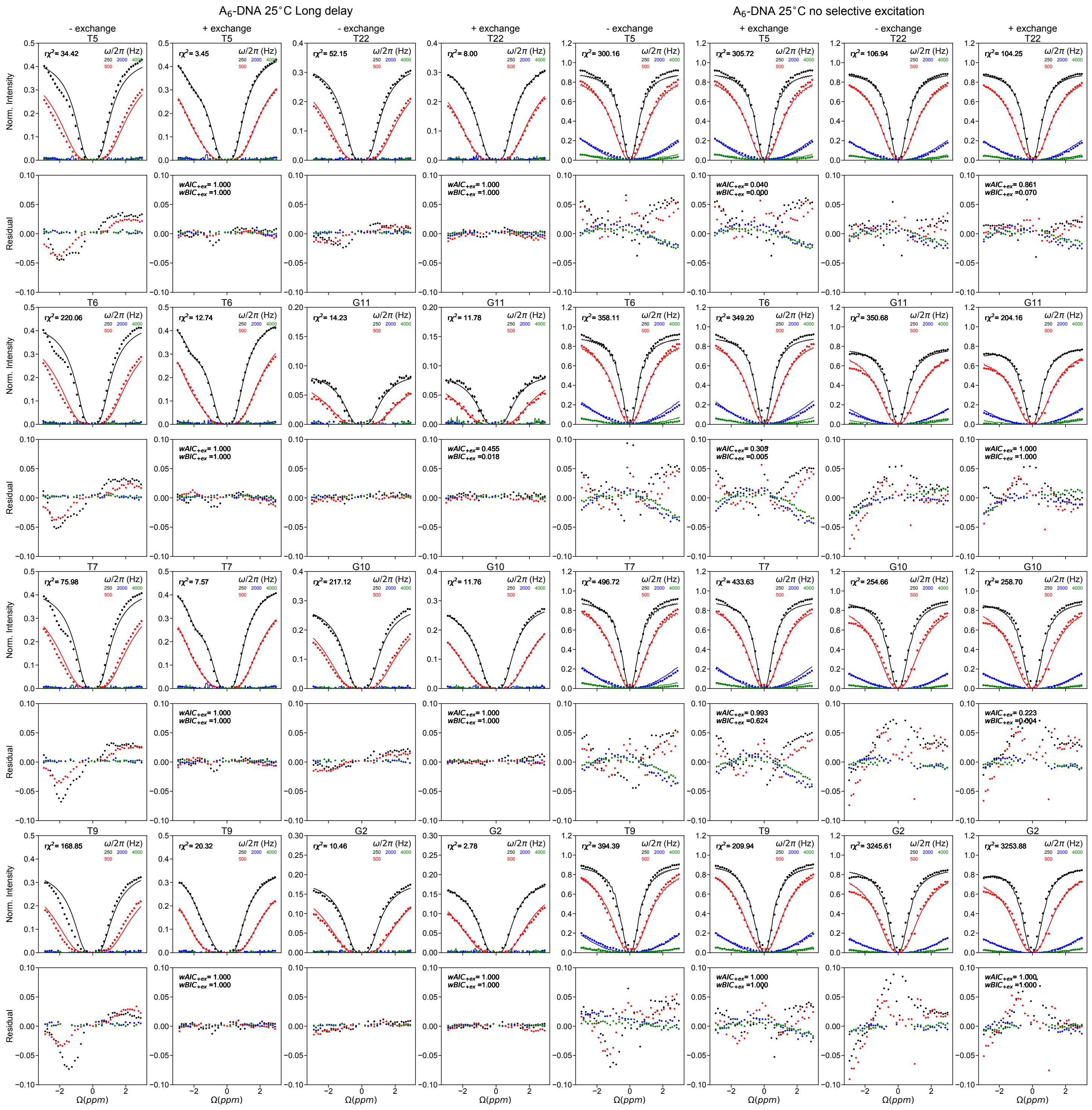

Fig. S4. ^{1}H CEST profiles for A_6-DNA (pH 6.8) at 25°C with long relaxation delay (400 ms) and using non-selective excitation of imino protons. RF field powers used for ^{1}H CEST are color-coded. Shown are the ^{1}H CEST data fits to the Bloch-McConnell equations with and without (k_{ex} = $\Delta\omega$ = p_{ES} = 0) 2-state chemical exchange. Shown below the CEST profiles are residual plots (normalized intensity - fitted normalized intensity), computed as described in Methods. Reduced chi-square ($r\chi^2$), Akaike's

(w*AIC*) and Bayesian (w*BIC*) information criterion weights were used to select the model with (+ex) or without (-ex) exchange, as described in the main text. Error bars for ^{1}H CEST profiles (typically smaller than data points) were obtained using the standard deviation of 3 measurements of peak intensity with zero relaxation delay as described in Methods.

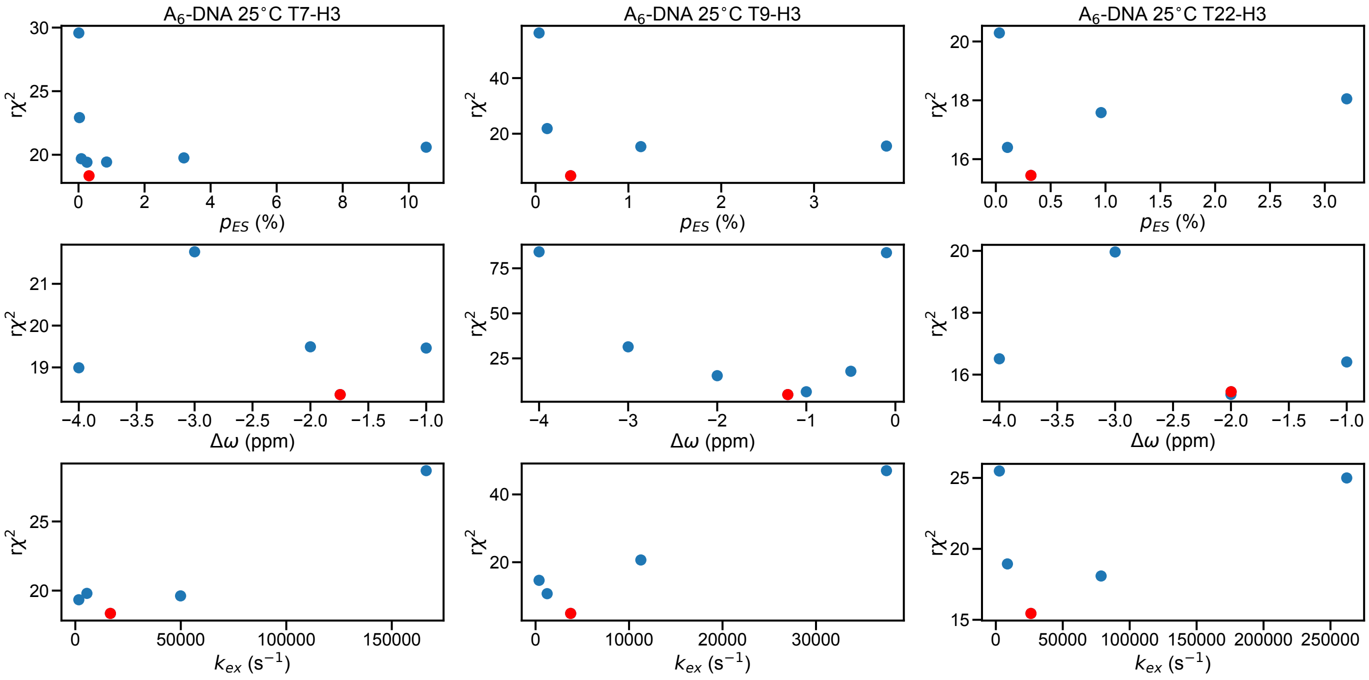

Fig. S5. Degeneracy analysis for representative ^{1}H CEST profiles. Shown is the quality of a 2-state fit ($r\chi^2$) to the ^{1}H CEST profile measured for T7-H3, T9-H3 and T22-H3 in A_6-DNA as a function of individually holding each exchange parameter p_{ES}, Δω and k_{ex} to a different value while allowing all other exchange parameters to float during fit. The best-fit exchange parameters (p_{ES}, Δω and k_{ex}) are in red.

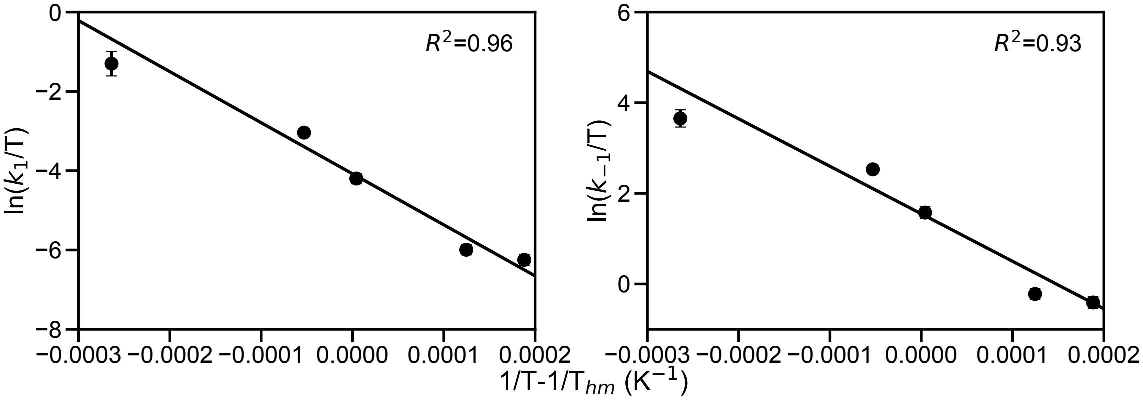

Fig. S6. The temperature dependence of k_1 and k_{-1} for A6-T9 Watson-Crick to Hoogsteen exchange in A_6-DNA. R^2 denotes coefficient of determination (see Methods). Error bars were determined by propagating the errors in k_1 and k_{-1} obtained from 2-state fits of the ^{1}H CEST profile for T9-H3 to the Bloch-McConnell equations (Methods).

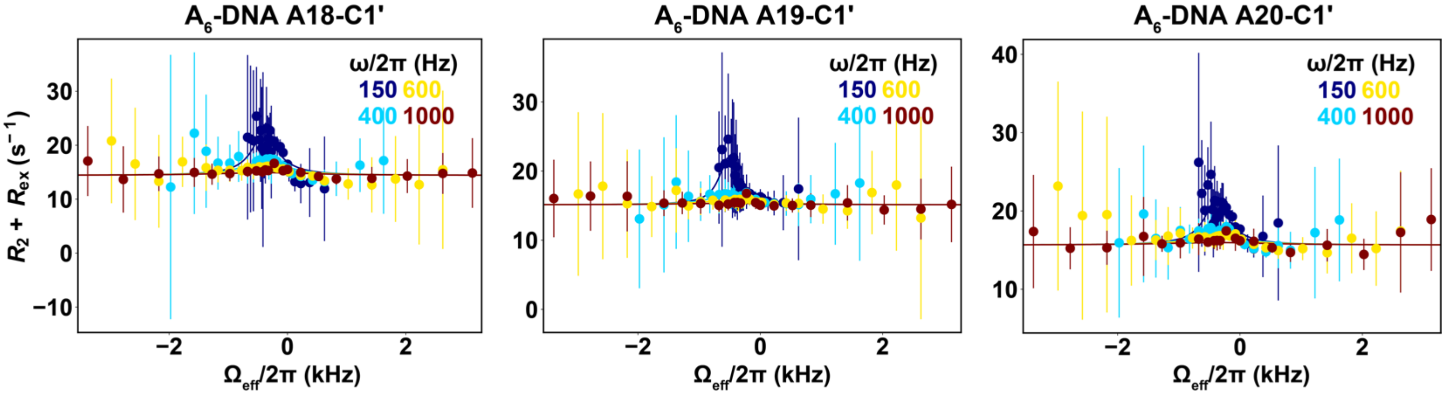

Fig. S7. Off-resonance $R_{1\rho}$ profiles measured for A-tract residues A18, A19 and A20 in A_6-DNA. Spin-lock powers are color coded. Error bars in the $R_{1\rho}$ profiles were obtained using Monte-Carlo simulations (Rangadurai et al., 2019). Solid lines denote a fit of the data (points) to the Bloch-McConnell equations assuming a 2-state exchange model.

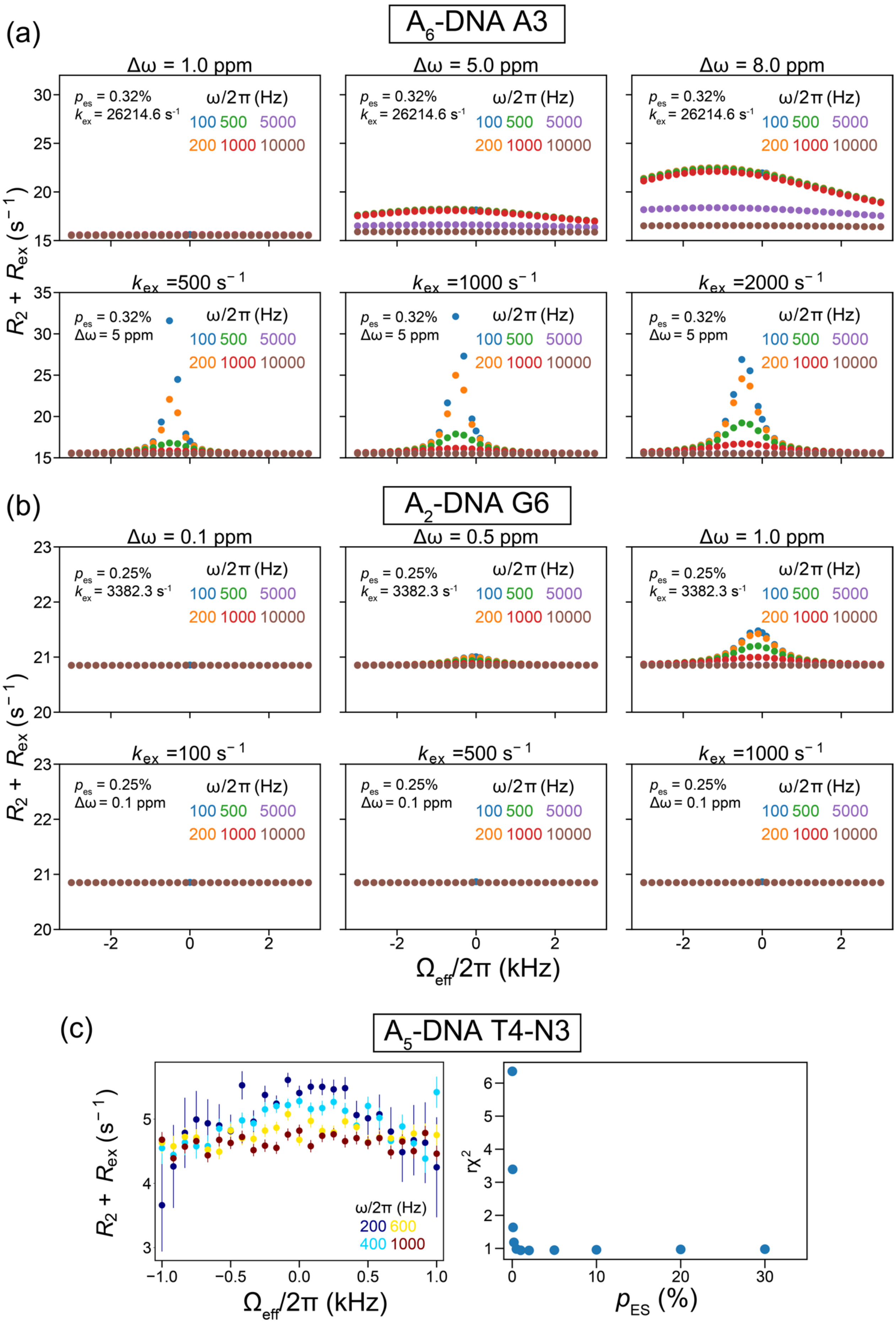
(a)
A6-DNA A3
Δω = 1.0 ppm
Δω = 5.0 ppm
Δω = 8.0 ppm
pes = 0.32%
kex = 26214.6 s-1
ω/2π (Hz)
100 500 5000
200 1000 10000
kex =500 s−1
kex =1000 s−1
kex =2000 s−1
pes = 0.32%
Δω = 5 ppm
R2 + Rex (s−1)
(b)
A2-DNA G6
Δω = 0.1 ppm
Δω = 0.5 ppm
Δω = 1.0 ppm
pes = 0.25%
kex = 3382.3 s-1
kex =100 s−1
kex =500 s−1
kex =1000 s−1
pes = 0.25%
Δω = 0.1 ppm
Ωeff/2π (kHz)
(c)
A5-DNA T4-N3
ω/2π (Hz)
200 600
400 1000
Ωeff/2π (kHz)
rχ2
pES (%)

Fig. S8. Degeneracy analysis of simulated off-resonance ^{13}C $R_{1\rho}$ data. (a) Simulated off-resonance ^{13}C $R_{1\rho}$ profiles for A3-C1' in A_6-DNA for varying Δω and k_{ex}. p_{ES} = 0.32% and k_{ex} = 26214.6 s^{-1} were obtained from fitting of the ^1H CEST profile for T22-H3 (Table S1). Simulations assumed a longitudinal relaxation rate constant R_1 = 1.61 s^{-1} and a transverse relaxation rate constant R_2 = 15.51 s^{-1} from Shi *et al* (Shi et al., 2018). RD signals were substantial when Δω = 5 ppm, or when k_{ex} is small (k_{ex} = 500 – 2,000 s^{-1}) indicating that the lack of ^{13}C RD is likely due to fast exchange and/or small Δω. (b) Simulated ^{13}C $R_{1\rho}$ profile for G6-C1' in A_2-DNA. p_{ES} = 0.25% and k_{ex} = 3382.3 s^{-1} were obtained from the ^1H CEST data (Table S1). R_1 and R_2 were assumed to be 1.21 s^{-1} and 20.85 s^{-1} respectively, and were derived from Shi *et al* (Shi et al., 2018). RD signals were substantial when Δω = 1 ppm, indicating that the lack of ^{13}C RD is most likely due to a small Δω value. (c) The simulated ^{15}N $R_{1\rho}$ profile (left) for T4-N3 in A_5-DNA assuming p_{ES} = 2.73%, k_{ex} = 2724 s^{-1}, Δω = -0.99 ppm, R_1 = 1.28 s^{-1}, and R_2 = 4.44 s^{-1} obtained from Alvey *et al* (Alvey et al., 2014). Error bars (2%) were simulated using a Monte-Carlo scheme (Rangadurai et al., 2019). Shown on the right is $r\chi^2$ as a function of varying p_{ES}.

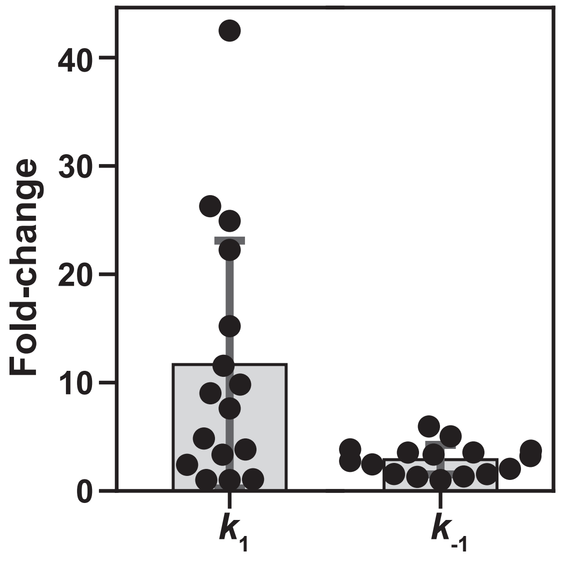

Fig. S9. Variation of k_1 and k_{-1} for the Watson-Crick to Hoogsteen exchange for different bps. Shown are the forward (k_1) and backward (k_{-1}) rate constants for A-T and G-C bps in A_6-DNA and A_2-DNA at 25 °C and for A_5-DNA at 26 °C. The values shown are calculated as $k_1/k_{1,min}$ or $k_{-1}/k_{-1,min}$, where $k_{1,min}$ and $k_{1,min}$ are the smallest k_1 and k_{-1} values respectively. All rate constants are provided in Table S1. Error bars are the standard deviations across all data points in each group.

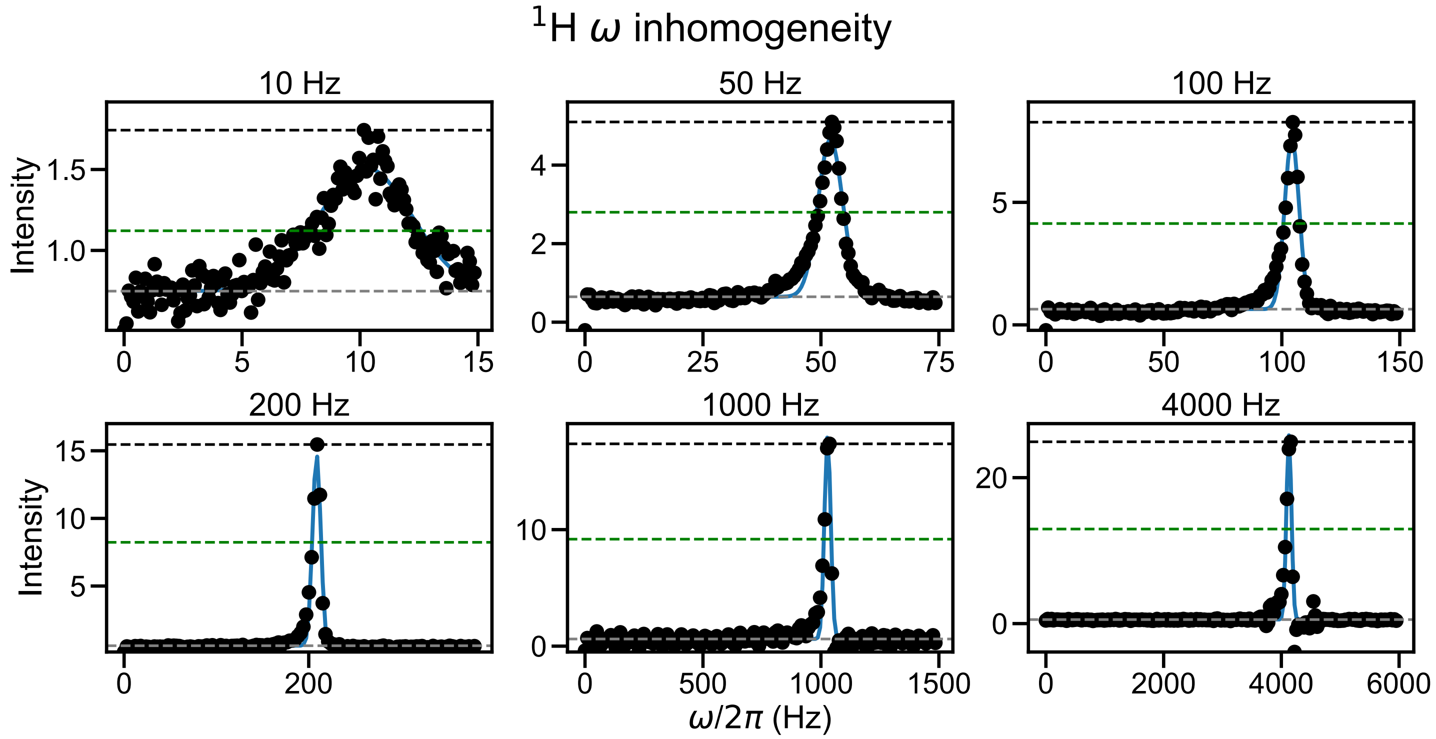

Fig. S10. Distributions of the RF field strength ω/2π (Hz) for ^{1}H spins. The applied apparent RF power is indicated in Hz (10 – 4000 Hz). Spectral baselines, half maximum heights, and the maxima are highlighted using dashed gray lines, dashed green lines, and dashed black lines, respectively. The data were fit (solid blue lines) to a gaussian function to extract the baseline value and the standard deviation (σ).

Supplementary tables

Table S1. Exchange parameters obtained from 2-state fitting of ^{1}H CEST and off-resonance ^{13}C $R_{1\rho}$ data

^{1}H CEST							
Sample	Resonance	Δω (ppm)	p_{ES} (%)	k_{ex} (s^{-1})	R_1 (s^{-1})	R_2 (s^{-1})	$r\chi^2$
A_6-DNA 5°C pH 6.8 T_{EX} = 100 ms	T5-H3	-0.55±0.06	0.12±0.15	255.8±411.7	3.46±0.01	29.52±0.28	10.2
	T9-H3	-1.19±0.04	0.29±0.01	185.4±25.0	3.38±0.01	25.94±0.41	30.4
A_6-DNA 10°C pH 6.8 T_{EX} = 100 ms	T9-H3	-1.19±0.04	0.31±0.01	227.8±28.0	2.99±0.01	21.59±0.09	8.4
	T22-H3	-2.71±0.20	0.02±0.01	2919.7±1559.6	3.00±0.01	20.91±0.22	50.9
A_6-DNA 20°C pH 6.8 T_{EX} = 100 ms	T9-H3	-1.22±0.02	0.29±0.05	1479.1±200.8	2.73±0.02	16.37±0.34	186.7
	T22-H3	-1.97±0.42	0.16±0.04	12215.5±2333.0	2.78±0.02	12.91±0.63	99.7
A_6-DNA 25°C pH 6.8 T_{EX} = 100 ms	T5-H3	-2.00±0.11	0.06±0.01	1569.0±640.3	1.97±0.02	13.32±0.31	54.0
	T6-H3	-2.20±0.11	0.06±0.02	1754.1±611.8	2.02±0.01	15.18±0.11	96.2
	T7-H3	-1.87±0.08	0.09±0.03	1413.6±628.2	2.05±0.02	13.45±0.27	19.3
	T9-H3	-1.21±0.03	0.38±0.02	3753.9±172.7	2.73±0.01	15.58±0.08	4.9
	G10-H1	-1.97±0.20	0.03±0.01	2700.1±1165.1	3.30±0.02	17.23±0.08	16.9
	G10-H1*	-1.50±0.55	0.03±0.02	5616.3±2960.9	3.34±0.01	16.26±0.10	27.9
	T22-H3	-2.00±0.41	0.32±0.07	26214.6±4290.4	2.87±0.02	9.28±0.62	15.5
A_6-DNA 25°C pH 6.8 T_{EX} = 400 ms	G2-H1	-2.42±0.19	0.05±0.01	5977.2±1582.2	4.14±0.02	15.61±0.56	2.8
	T5-H3	-2.13±0.04	0.07±0.01	2780.8±319.0	1.88±0.02	13.73±0.17	3.5
	T6-H3	-2.21±0.02	0.08±0.01	2244.7±142.6	1.89±0.01	13.72±0.17	12.7
	T7-H3	-2.00±0.03	0.10±0.01	1931.4±176.3	1.96±0.02	14.01±0.21	7.6
	T9-H3	-1.52±0.09	0.25±0.07	3561.5±511.6	2.55±0.03	14.72±0.57	20.3
	G10-H1	-2.47±0.10	0.05±0.01	5922.7±1195.7	3.04±0.02	15.13±0.33	11.8
	T22-H3	-2.7±0.2	0.08±0.02	10688.5±1716.3	2.68±0.01	13.45±0.63	8.0
A_6-DNA 30°C pH 6.8 T_{EX} = 100 ms	G2-H1	-3.34±4.06	0.55±0.66	68316.0±54714.9	6.06±0.02	5.07±5.67	29.6
	T4-H3	-1.60±0.03	0.29±0.02	4096.7±264.2	2.54±0.02	11.49±0.28	46.1
	T5-H3	-1.70±0.06	0.18±0.02	4452.4±528.5	1.87±0.02	11.95±0.41	31.0
	T6-H3	-2.07±0.04	0.13±0.01	2974.1±278.3	2.03±0.02	12.27±0.22	14.8
	T7-H3	-1.78±0.05	0.20±0.02	3472.5±314.9	2.13±0.02	12.63±0.45	31.1
	G10-H1	-2.11±0.20	0.07±0.02	5498.5±1515.4	3.26±0.02	13.11±0.59	21.0
	T22-H3	-1.68±0.53	0.32±0.10	27127.5±6510.8	3.59±0.02	11.61±0.61	56.8
A_6-DNA 45°C pH 6.8 T_{EX} = 80 ms	T5-H3	-1.83±0.21	0.19±0.06	10889.3±1837.5	11.24±0.06	18.65±0.47	6.0
	T6-H3	-2.06±0.13	0.25±0.03	9594.5±985.2	11.41±0.04	18.42±0.46	10.0
	T9-H3	-0.90±0.17	0.70±0.17	12362.5±2348.9	14.25±0.05	19.72±0.56	2.5
A_2-DNA 25°C pH 5.4 T_{EX} = 100 ms	G6-H1	-1.39±0.07	0.25±0.04	3382.3±555.6	7.34±0.03	19.23±0.77	20.1
	T8-H3	-1.60±0.02	0.65±0.07	3596.8±193.0	2.22±0.02	9.93±0.72	29.9
	T9-H3	-1.16±0.05	0.78±0.14	5110.6±429.6	2.96±0.02	15.66±0.96	10.6
	G10-H1	-2.00±0.02	0.84±0.25	2484.5±118.7	7.74±0.04	17.08±0.83	12.0
	T18-H3	-1.64±0.05	0.41±0.05	6013.3±485.7	5.35±0.02	15.91±0.77	9.5
	G20-H1	-1.46±0.09	0.28±0.06	3870.7±813.1	6.69±0.04	17.09±1.23	53.6
	G23-H1	-1.41±0.12	0.26±0.08	5246.0±1262.4	12.48±0.04	26.13±1.23	31.3
A_5-DNA 26°C pH 5.2 T_{EX} = 100 ms	T4-H3	-1.93±0.06	0.20±0.03	3570.8±398.1	2.34±0.03	12.07±0.56	4.5
	T5-H3	-2.28±0.09	0.14±0.02	3241.6±506.5	1.95±0.03	12.49±0.68	13.0
	T6-H3	-2.30±0.05	0.11±0.02	2054.8±482.7	2.18±0.02	13.22±0.26	62.2
	T7-H3	-2.04±0.03	0.23±0.05	1559.6±208.1	2.14±0.03	12.62±0.44	4.0
	T8-H3	-1.94±0.03	0.33±0.07	2790.3±273.3	3.61±0.03	15.24±0.52	2.2
	T22-H3	-1.58±0.54	0.03±0.03	5440.7±4846.8	3.00±0.02	17.79±0.57	4.7
	G16-H1	-1.97±0.12	0.24±0.18	1307.0±807.9	9.28±0.09	28.32±1.33	28.3
^{13}C $R_{1\rho}$							
A_6-DNA 25°C pH 6.8	A18-C1'	2.56±0.17	0.27±0.03	1303.6±225.5	2.20±0.06	14.41±0.14	0.3
	A19-C1'	3.44±0.15	0.13±0.04	637.2±218.2	2.13±0.03	15.12±0.05	0.1
	A20-C1'	2.56±0.18	0.19±0.02	1181.0±212.6	2.10±0.05	15.65±0.11	0.3

(* results obtained from the experiment where only G10 and G2 in A6-DNA were selective excited).

Table S2. RF field powers and offsets used in ^{1}H CEST experiments.

**Sample**	**[RF field power] {offset frequencies}**
	**[$\omega/2\pi$ (Hz)] {$\Omega/2\pi$ (Hz)}**
A$_6$-DNA 5°C<br>pH 6.8<br>T_{EX} = 100 ms	[10] {-4481, -4224, -3967, -3710, -3453, -3196, -2939, -2682, -2620, -2558, -2496, -2434, -2372, -2310, -2248, -2186, -2124, -2062, -2000, -1938, -1876, -1814, -1752, -1690, -1628, -1566, -1504, -1442, -1380, -1318, -1256, -1194, -1132, -1070, -1008, -945, -883, -820, -757, -694, -631, -568, -505, -442, -378, -315, -252, -189, -126, -63, 0, 62, 125, 189, 252, 315, 377, 439, 501, 563, 625, 687, 749, 811, 873, 935, 997, 1059, 1121, 1183, 1245, 1307, 1369, 1431, 1493, 1556, 1618, 1680, 1742, 1804, 1866, 1928, 1990, 2052, 2114, 2371, 2628, 2885, 3142, 3399, 3656, 3913}<br>[50] {-4481, -4224, -3967, -3710, -3453, -3196, -2939, -2682, -2620, -2558, -2496, -2434, -2372, -2310, -2248, -2186, -2124, -2062, -2000, -1938, -1876, -1814, -1752, -1690, -1628, -1566, -1504, -1442, -1380, -1318, -1256, -1194, -1132, -1070, -1008, -946, -883, -820, -757, -694, -631, -568, -505, -442, -378, -315, -252, -189, -126, -63, 62, 125, 189, 252, 315, 377, 439, 501, 563, 625, 687, 749, 811, 873, 935, 997, 1059, 1121, 1183, 1245, 1307, 1369, 1431, 1493, 1556, 1618, 1680, 1742, 1804, 1866, 1928, 1990, 2052, 2114, 2371, 2628, 2885, 3142, 3399, 3656, 3913}<br>[100] {-4481, -4224, -3967, -3710, -3453, -3196, -2939, -2682, -2620, -2558, -2496, -2434, -2372, -2310, -2248, -2186, -2124, -2062, -2000, -1938, -1876, -1814, -1752, -1690, -1628, -1566, -1504, -1442, -1380, -1318, -1256, -1194, -1132, -1070, -1008, -946, -883, -820, -757, -694, -631, -568, -505, -442, -379, -315, -252, -189, -126, 62, 125, 189, 252, 315, 377, 439, 501, 563, 625, 687, 749, 811, 873, 935, 997, 1059, 1121, 1183, 1245, 1307, 1369, 1431, 1493, 1556, 1618, 1680, 1742, 1804, 1866, 1928, 1990, 2052, 2114, 2371, 2628, 2885, 3142, 3399, 3656, 3913}<br>[200] {-4481, -4224, -3967, -3710, -3453, -3196, -2939, -2682, -2620, -2558, -2496, -2434, -2372, -2310, -2248, -2186, -2124, -2062, -2000, -1938, -1876, -1814, -1752, -1690, -1628, -1566, -1504, -1442, -1380, -1318, -1256, -1194, -1132, -1070, -1008, -946, -883, -820, -757, -694, -631, -568, -505, -442, -379, -315, -252, -189, -126, -63, 62, 125, 189, 252, 315, 377, 439, 501, 563, 625, 687, 749, 811, 873, 935, 997, 1059, 1121, 1183, 1245, 1307, 1369, 1431, 1493, 1556, 1618, 1680, 1742, 1804, 1866, 1928, 1990, 2052, 2114, 2371, 2628, 2885, 3142, 3399, 3656, 3913}
A$_6$-DNA 10°C<br>pH 6.8<br>T_{EX} = 100 ms	[10] {-4050, -3810, -3570, -3330, -3090, -2850, -2765, -2679, -2593, -2508, -2422, -2336, -2251, -2165, -2079, -1994, -1908, -1822, -1737, -1651, -1565, -1480, -1394, -1308, -1223, -1137, -1051, -942, -833, -724, -615, -506, -397, -288, -179, -70, 38, 147, 233, 318, 404, 490, 575, 661, 747, 832, 918, 1004, 1089, 1175, 1261, 1346, 1432, 1518, 1603, 1689, 1775, 1860, 1946, 2186, 2426, 2666, 2906, 3145}<br>[50] {-4049, -3810, -3570, -3330, -3090, -2850, -2764, -2679, -2593, -2507, -2422, -2336, -2250, -2165, -2079, -1994, -1908, -1822, -1737, -1651, -1565, -1480, -1394, -1308, -1223, -1137, -1051, -942, -833, -724, -615, -506, -397, -288, -179, -70, 38, 147, 233, 318, 404, 490, 575, 661, 747, 832, 918, 1004, 1089, 1175, 1261, 1346, 1432, 1518, 1603, 1689, 1775, 1860, 1946, 2186, 2426, 2666, 2906, 3145}<br>[250] {-4050, -3810, -3570, -3330, -3090, -2850, -2765, -2679, -2593, -2508, -2422, -2336, -2251, -2165, -2079, -1994, -1908, -1822, -1737, -1651, -1565, -1480, -1394, -1308, -1223, -1137, -1051, -942, -833, -724, -615, -506, -397, -288, -179, -70, 147, 233, 318, 404, 490, 575, 661, 747, 832, 918, 1004, 1089, 1175, 1261, 1346, 1432, 1518, 1603, 1689, 1775, 1860, 1946, 2186, 2426, 2666, 2906, 3145}<br>[500] {-4049, -3810, -3570, -3330, -3090, -2850, -2765, -2679, -2593, -2508, -2422, -2336, -2251, -2165, -2079, -1994, -1908, -1822, -1737, -1651, -1565, -1480, -1394, -1308, -1223, -1137, -1051, -942, -833, -724, -615, -506, -397, -288, -179, -70, 38, 147, 233, 318, 404, 490, 575, 661, 747, 832, 918, 1004, 1089, 1175, 1261, 1346, 1432, 1518, 1603, 1689, 1775, 1860, 1946, 2186, 2426, 2666, 2906, 3145}<br>[1000] {-4050, -3810, -3570, -3330, -3090, -2850, -2765, -2679, -2593, -2508, -2422, -2336, -2251, -2165, -2079, -1994, -1908, -1822, -1737, -1651, -1565, -1480, -1394, -1308, -1223, -1137, -1051, -942, -833, -724, -615, -506, -397, -288, 38, 147, 318, 404, 490, 575, 661, 747, 832, 918, 1004, 1089, 1175, 1261, 1346, 1432, 1518, 1603, 1689, 1775, 1860, 1946, 2186, 2426, 2666, 2906, 3145}
A$_6$-DNA 20°C<br>pH 6.8<br>T_{EX} = 100 ms	[100] {-4476, -4219, -3962, -3705, -3448, -3191, -2934, -2677, -2615, -2553, -2491, -2429, -2367, -2305, -2242, -2180, -2118, -2056, -1994, -1932, -1870, -1808, -1746, -1684, -1622, -1560, -1498, -1436, -1374, -1312, -1250, -1188, -1126, -1064, -1002, -940, -878, -815, -751, -688, -625, -562, -499, -436, -373, -310, -247, -183, -120, -57, 68, 131, 194, 257, 321, 383, 445, 507, 569, 631, 693, 755, 817, 879, 941, 1003, 1065, 1127, 1189, 1251, 1313, 1375, 1437, 1499, 1561, 1623, 1685, 1747, 1809, 1871, 1933, 1996, 2058, 2120, 2377, 2634, 2891, 3148, 3405, 3662, 3919}

	[250] {-4476, -4219, -3962, -3705, -3448, -3191, -2934, -2677, -2615, -2553, -2491, -2429, -2366, -2304, -2242, -2180, -2118, -2056, -1994, -1932, -1870, -1808, -1746, -1684, -1622, -1560, -1498, -1436, -1374, -1312, -1250, -1188, -1126, -1064, -1002, -940, -878, -815, -751, -688, -625, -562, -499, -436, -373, -310, -246, -183, -120, 5, 68, 131, 194, 258, 321, 383, 445, 507, 569, 631, 693, 755, 817, 879, 941, 1003, 1065, 1127, 1189, 1251, 1313, 1375, 1437, 1499, 1561, 1623, 1685, 1747, 1809, 1871, 1934, 1996, 2058, 2120, 2377, 2634, 2891, 3148, 3405, 3662, 3919}<br>[500] {-4476, -4219, -3962, -3705, -3448, -3191, -2934, -2677, -2615, -2553, -2491, -2429, -2366, -2304, -2242, -2180, -2118, -2056, -1994, -1932, -1870, -1808, -1746, -1684, -1622, -1560, -1498, -1436, -1374, -1312, -1250, -1188, -1126, -1064, -1002, -940, -878, -815, -751, -688, -625, -562, -499, -436, -373, -310, -246, -183, -120, 5, 131, 194, 258, 321, 383, 445, 507, 569, 631, 693, 755, 817, 879, 941, 1003, 1065, 1127, 1189, 1251, 1313, 1375, 1437, 1499, 1561, 1623, 1685, 1747, 1809, 1871, 1934, 1996, 2058, 2120, 2377, 2634, 2891, 3148, 3405, 3662, 3919}
A_6-DNA 25°C<br>pH 6.8<br>T_{EX} = 100 ms	[250] {-6424, -6167, -5910, -5653, -5396, -5139, -4882, -4625, -4368, -4111, -3854, -3597, -3340, -3083, -2826, -2748, -2670, -2591, -2513, -2435, -2357, -2279, -2200, -2122, -2044, -1966, -1887, -1809, -1731, -1653, -1575, -1496, -1418, -1340, -1262, -1184, -1105, -1027, -918, -809, -700, -591, -482, -373, -264, -155, 171, 249, 328, 406, 484, 562, 641, 719, 797, 875, 953, 1032, 1110, 1188, 1266, 1344, 1423, 1501, 1579, 1657, 1736, 1814, 1892, 1970, 2227, 2484, 2741, 2998, 3255, 3512, 3769, 4026, 4283, 4540, 4797, 5054, 5311, 5568}<br>[500] {-6410, -6153, -5896, -5639, -5382, -5125, -4868, -4611, -4354, -4097, -3840, -3583, -3326, -3069, -2812, -2733, -2655, -2577, -2499, -2421, -2342, -2264, -2186, -2108, -2030, -1951, -1873, -1795, -1717, -1638, -1560, -1482, -1404, -1326, -1247, -1169, -1091, -1013, -904, -795, -686, -577, -468, -359, -249, -140, -31, 77, 186, 264, 342, 420, 498, 577, 655, 733, 811, 890, 968, 1046, 1124, 1202, 1281, 1359, 1437, 1515, 1594, 1672, 1750, 1828, 1906, 1985, 2242, 2499, 2756, 3013, 3270, 3527, 3784, 4041, 4298, 4555, 4812, 5069, 5326, 5583}<br>[2000] {-6410, -6153, -5896, -5639, -5382, -5125, -4868, -4611, -4354, -4097, -3840, -3583, -3326, -3069, -2812, -2733, -2655, -2577, -2499, -2421, -2342, -2264, -2186, -2108, -2029, -1951, -1873, -1795, -1717, -1638, -1560, -1482, -1404, -1326, -1247, -1169, -1091, -1013, -904, -795, -686, -577, -468, -359, -31, 77, 186, 342, 420, 499, 577, 655, 733, 811, 890, 968, 1046, 1124, 1202, 1281, 1359, 1437, 1515, 1594, 1672, 1750, 1828, 1906, 1985, 2242, 2499, 2756, 3013, 3270, 3527, 3784, 4041, 4298, 4555, 4812, 5069, 5326, 5583}<br>[4000] {-6410, -6153, -5896, -5639, -5382, -5125, -4868, -4611, -4354, -4097, -3840, -3583, -3326, -3069, -2812, -2733, -2655, -2577, -2499, -2421, -2342, -2264, -2186, -2108, -2030, -1951, -1873, -1795, -1717, -1638, -1560, -1482, -1404, -1326, -1247, -1169, -1091, -1013, -904, -795, -686, -577, -359, -140, -31, 264, 342, 498, 577, 655, 733, 811, 890, 968, 1046, 1124, 1202, 1281, 1359, 1437, 1515, 1594, 1672, 1750, 1828, 1906, 1985, 2242, 2499, 2756, 3013, 3270, 3527, 3784, 4041, 4298, 4555, 4812, 5069, 5326, 5583}
A_6-DNA 25°C<br>pH 6.8<br>T_{EX} = 400 ms	[250] {-6453, -6196, -5939, -5682, -5425, -5168, -4911, -4654, -4397, -4140, -3883, -3626, -3369, -3112, -2855, -2777, -2698, -2620, -2542, -2464, -2386, -2307, -2229, -2151, -2073, -1995, -1916, -1838, -1760, -1682, -1603, -1525, -1447, -1369, -1291, -1212, -1134, -1056, -947, -838, -729, -620, -511, -402, -293, -184, -75, 33, 221, 299, 377, 455, 533, 612, 690, 768, 846, 925, 1003, 1081, 1159, 1237, 1316, 1394, 1472, 1550, 1629, 1707, 1785, 1863, 1941, 2198, 2455, 2712, 2969, 3226, 3483, 3740, 3997, 4254, 4511, 4768, 5025, 5282, 5539}<br>[500] {-6453, -6196, -5939, -5682, -5425, -5168, -4911, -4654, -4397, -4140, -3883, -3626, -3369, -3112, -2855, -2777, -2698, -2620, -2542, -2464, -2386, -2307, -2229, -2151, -2073, -1995, -1916, -1838, -1760, -1682, -1603, -1525, -1447, -1369, -1291, -1212, -1134, -1056, -947, -838, -729, -620, -511, -402, -293, 221, 299, 377, 455, 533, 612, 690, 768, 846, 925, 1003, 1081, 1159, 1237, 1316, 1394, 1472, 1550, 1629, 1707, 1785, 1863, 1941, 2198, 2455, 2712, 2969, 3226, 3483, 3740, 3997, 4254, 4511, 4768, 5025, 5282, 5539}<br>[2000] {-6453, -6196, -5939, -5682, -5425, -5168, -4911, -4654, -4397, -4140, -3883, -3626, -3369, -3112, -2855, -2777, -2698, -2620, -2542, -2464, -2386, -2307, -2229, -2151, -2073, -1994, -1916, -1838, -1760, -1682, -1603, -1525, -1447, -1369, -1291, -1212, -1134, -1056, -947, -838, -729, -402, -75, 299, 612, 768, 846, 1081, 1159, 1237, 1316, 1394, 1472, 1550, 1629, 1707, 1785, 1863, 1941, 2198, 2455, 2712, 2969, 3226, 3483, 3740, 3997, 4254, 4511, 4768, 5025, 5282, 5539}<br>[4000] {-6453, -6196, -5939, -5682, -5425, -5168, -4911, -4654, -4397, -4140, -3883, -3626, -3369, -3112, -2855, -2777, -2698, -2620, -2542, -2464, -2386, -2307, -2229, -2151, -2073, -1995, -1760, -1525, -1369, -1212, -838, -511, -293, -184, -75, 142, 299, 377, 533, 612, 768, 925, 1003, 1081, 1316, 1394, 1707, 1785, 1863, 1941, 2198, 2455, 2712, 2969, 3226, 3483, 3740, 3997, 4254, 4511, 4768, 5025, 5282, 5539}
A_6-DNA 30°C<br>pH 6.8<br>T_{EX} = 100 ms	[100] {-4031, -3791, -3551, -3311, -3071, -2831, -2756, -2681, -2606, -2531, -2456, -2382, -2307, -2232, -2157, -2082, -2007, -1932, -1857, -1782, -1707, -1632, -1557, -1482, -1407, -1332, -1257, -1182, -1107, -1032, -947, -861, -775, -690, -604, -518, -433, -347, -261, -176, -90, -4, 80, 166,

	241, 316, 391, 466, 541, 616, 691, 766, 841, 916, 991, 1066, 1140, 1215, 1290, 1365, 1440, 1515, 1590, 1665, 1740, 1815, 1890, 1965, 2205, 2445, 2685, 2924, 3164}<br>[250] {-4031, -3791, -3551, -3311, -3071, -2831, -2756, -2681, -2606, -2531, -2456, -2382, -2307, -2232, -2157, -2082, -2007, -1932, -1857, -1782, -1707, -1632, -1557, -1482, -1407, -1332, -1257, -1182, -1107, -1032, -947, -861, -775, -690, -604, -518, -433, -347, -261, -176, 166, 241, 316, 391, 466, 541, 616, 691, 766, 841, 916, 991, 1066, 1140, 1215, 1290, 1365, 1440, 1515, 1590, 1665, 1740, 1815, 1890, 1965, 2205, 2445, 2685, 2924, 3164}<br>[500] {-4031, -3791, -3551, -3311, -3071, -2831, -2756, -2681, -2606, -2531, -2456, -2381, -2307, -2232, -2157, -2082, -2007, -1932, -1857, -1782, -1707, -1632, -1557, -1482, -1407, -1332, -1257, -1182, -1107, -1032, -947, -861, -775, -690, -604, -518, -433, -347, -261, -176, -90, 80, 166, 241, 316, 391, 466, 541, 616, 691, 766, 841, 916, 991, 1066, 1141, 1215, 1290, 1365, 1440, 1515, 1590, 1665, 1740, 1815, 1890, 1965, 2205, 2445, 2685, 2925, 3164}<br>[750] {-4031, -3791, -3551, -3311, -3071, -2831, -2756, -2681, -2606, -2531, -2456, -2382, -2307, -2232, -2157, -2082, -2007, -1932, -1857, -1782, -1707, -1632, -1557, -1482, -1407, -1332, -1257, -1182, -1107, -1032, -947, -861, -775, -690, -604, -518, -433, -347, -261, -90, 166, 241, 316, 391, 466, 541, 616, 691, 766, 841, 916, 991, 1066, 1140, 1215, 1290, 1365, 1440, 1515, 1590, 1665, 1740, 1815, 1890, 1965, 2205, 2445, 2685, 2924, 3164}
A_6-DNA 45°C<br>pH 6.8<br>T_{EX} = 80 ms	[10] {-6476, -6219, -5962, -5705, -5448, -5191, -4934, -4678, -4421, -4164, -3907, -3650, -3393, -3136, -2879, -2800, -2722, -2644, -2566, -2487, -2409, -2331, -2253, -2175, -2096, -2018, -1940, -1862, -1783, -1705, -1627, -1549, -1471, -1392, -1314, -1236, -1158, -1080, -971, -861, -752, -643, -534, -425, -316, -207, -98, 10, 119, 197, 275, 353, 432, 510, 588, 666, 745, 823, 901, 979, 1057, 1136, 1214, 1292, 1370, 1448, 1527, 1605, 1683, 1761, 1840, 1918, 2175, 2432, 2689, 2946, 3203, 3460, 3717, 3974, 4231, 4488, 4745, 5002, 5259, 5516}<br>[50] {-6477, -6220, -5963, -5706, -5449, -5192, -4935, -4678, -4421, -4164, -3907, -3650, -3393, -3136, -2879, -2801, -2723, -2645, -2566, -2488, -2410, -2332, -2253, -2175, -2097, -2019, -1941, -1862, -1784, -1706, -1628, -1549, -1471, -1393, -1315, -1237, -1158, -1080, -971, -862, -753, -644, -535, -426, -317, -208, -99, 118, 196, 275, 353, 431, 509, 587, 666, 744, 822, 900, 979, 1057, 1135, 1213, 1291, 1370, 1448, 1526, 1604, 1682, 1761, 1839, 1917, 2174, 2431, 2688, 2945, 3202, 3459, 3716, 3973, 4230, 4487, 4744, 5001, 5258, 5515}<br>[100] {-6477, -6220, -5963, -5706, -5449, -5192, -4935, -4678, -4421, -4164, -3907, -3650, -3393, -3136, -2879, -2800, -2722, -2644, -2566, -2488, -2409, -2331, -2253, -2175, -2096, -2018, -1940, -1862, -1784, -1705, -1627, -1549, -1471, -1392, -1314, -1236, -1158, -1080, -971, -862, -753, -644, -534, -425, -316, -207, -98, 119, 197, 275, 353, 432, 510, 588, 666, 744, 823, 901, 979, 1057, 1136, 1214, 1292, 1370, 1448, 1527, 1605, 1683, 1761, 1839, 1918, 2175, 2432, 2689, 2946, 3203, 3460, 3717, 3974, 4231, 4488, 4745, 5002, 5259, 5516}<br>[250] {-6477, -6220, -5963, -5706, -5449, -5192, -4935, -4678, -4421, -4164, -3907, -3650, -3393, -3136, -2879, -2801, -2722, -2644, -2566, -2488, -2410, -2331, -2253, -2175, -2097, -2019, -1940, -1862, -1784, -1706, -1627, -1549, -1471, -1393, -1315, -1236, -1158, -1080, -971, -862, -753, -644, -535, -426, -317, -208, -99, 197, 275, 353, 431, 509, 588, 666, 744, 822, 901, 979, 1057, 1135, 1213, 1292, 1370, 1448, 1526, 1605, 1683, 1761, 1839, 1917, 2174, 2431, 2688, 2945, 3202, 3459, 3716, 3973, 4230, 4487, 4744, 5001, 5258, 5515}<br>[1000] {-6477, -6220, -5963, -5706, -5449, -5192, -4935, -4678, -4421, -4164, -3907, -3650, -3393, -3136, -2879, -2801, -2722, -2644, -2566, -2488, -2409, -2331, -2253, -2175, -2097, -2018, -1940, -1862, -1784, -1705, -1627, -1549, -1471, -1393, -1314, -1236, -1158, -1080, -971, -862, -753, -644, -535, -426, -317, -99, 119, 197, 275, 353, 431, 510, 588, 666, 744, 823, 901, 979, 1057, 1135, 1214, 1292, 1370, 1448, 1526, 1605, 1683, 1761, 1839, 1918, 2175, 2432, 2689, 2946, 3203, 3460, 3717, 3974, 4231, 4488, 4745, 5001, 5258, 5515}<br>[2000] {-6477, -6220, -5963, -5706, -5449, -5192, -4935, -4678, -4421, -4164, -3907, -3650, -3393, -3136, -2879, -2801, -2722, -2644, -2566, -2488, -2410, -2331, -2253, -2175, -2097, -2018, -1940, -1862, -1784, -1706, -1627, -1549, -1471, -1393, -1315, -1236, -1158, -1080, -971, -862, -753, -644, -535, -426, 9, 275, 353, 431, 510, 588, 666, 744, 822, 901, 979, 1057, 1135, 1213, 1292, 1370, 1448, 1526, 1605, 1683, 1761, 1839, 1917, 2174, 2431, 2688, 2945, 3202, 3459, 3716, 3973, 4230, 4487, 4744, 5001, 5258, 5515}<br>[4000] {-6477, -6220, -5963, -5706, -5449, -5192, -4935, -4678, -4421, -4164, -3907, -3650, -3393, -3136, -2879, -2801, -2723, -2644, -2566, -2488, -2410, -2332, -2253, -2175, -2097, -2019, -1940, -1862, -1784, -1706, -1628, -1549, -1471, -1393, -1315, -1237, -1158, -1080, -971, -862, -753, -644, -535, -317, -208, 9, 353, 509, 588, 666, 744, 822, 900, 979, 1057, 1135, 1213, 1291, 1370, 1448, 1526, 1604, 1683, 1761, 1839, 1917, 2174, 2431, 2688, 2945, 3202, 3459, 3716, 3973, 4230, 4487, 4744, 5001, 5258, 5515}
A_2-DNA 25°C<br>pH 5.4	[100] {-4666, -4306, -3946, -3586, -3227, -2867, -2792, -2717, -2642, -2567, -2492, -2417, -2342, -2267, -2192, -2117, -2042, -1967, -1892, -1817, -1743, -1668, -1593, -1518, -1443, -1368, -1293,

T_{EX} = 100 ms	-1218, -1143, -1068, -982, -897, -811, -725, -640, -554, -468, -383, -297, -211, -126, -40, 45, 130, 205, 280, 355, 430, 505, 580, 655, 730, 805, 880, 955, 1030, 1105, 1180, 1255, 1330, 1405, 1480, 1555, 1630, 1705, 1779, 1854, 1929, 2289, 2649, 3009, 3369, 3728}<br>[250] {-4666, -4306, -3946, -3586, -3227, -2867, -2792, -2717, -2642, -2567, -2492, -2417, -2342, -2267, -2192, -2117, -2042, -1967, -1892, -1817, -1743, -1668, -1593, -1518, -1443, -1368, -1293, -1218, -1143, -1068, -982, -897, -811, -725, -640, -554, -468, -383, -297, -211, 45, 130, 205, 280, 355, 430, 505, 580, 655, 730, 805, 880, 955, 1030, 1105, 1180, 1255, 1330, 1405, 1480, 1555, 1630, 1705, 1779, 1854, 1929, 2289, 2649, 3009, 3369, 3728}<br>[500] {-4666, -4306, -3946, -3586, -3227, -2867, -2792, -2717, -2642, -2567, -2492, -2417, -2342, -2267, -2192, -2117, -2042, -1967, -1892, -1817, -1742, -1668, -1593, -1518, -1443, -1368, -1293, -1218, -1143, -1068, -982, -897, -811, -725, -640, -554, -468, -383, -297, -211, -40, 45, 130, 205, 280, 355, 430, 505, 580, 655, 730, 805, 880, 955, 1030, 1105, 1180, 1255, 1330, 1405, 1480, 1555, 1630, 1705, 1780, 1854, 1929, 2289, 2649, 3009, 3369, 3728}
A_5-DNA 25°C<br>pH 5.2<br>T_{EX} = 100 ms	[250] {-4491, -4234, -3977, -3720, -3463, -3206, -2949, -2692, -2630, -2568, -2506, -2444, -2382, -2320, -2258, -2196, -2134, -2072, -2010, -1947, -1885, -1823, -1761, -1699, -1637, -1575, -1513, -1451, -1389, -1327, -1265, -1203, -1141, -1079, -1017, -955, -893, -830, -767, -704, -640, -577, -514, -451, -388, -325, -262, -199, -135, -72, 53, 116, 179, 242, 305, 367, 429, 492, 554, 616, 678, 740, 802, 864, 926, 988, 1050, 1112, 1174, 1236, 1298, 1360, 1422, 1484, 1546, 1608, 1670, 1732, 1794, 1856, 1918, 1980, 2042, 2104, 2361, 2618, 2875, 3132, 3389, 3646, 3903}<br>[500] {-4464, -4207, -3950, -3693, -3436, -3179, -2922, -2665, -2603, -2541, -2479, -2417, -2355, -2293, -2231, -2169, -2107, -2045, -1983, -1921, -1859, -1797, -1735, -1672, -1610, -1548, -1486, -1424, -1362, -1300, -1238, -1176, -1114, -1052, -990, -928, -866, -803, -740, -677, -614, -550, -487, -424, -361, -298, -235, -172, -109, -45, 80, 206, 269, 332, 394, 456, 518, 580, 642, 704, 767, 829, 891, 953, 1015, 1077, 1139, 1201, 1263, 1325, 1387, 1449, 1511, 1573, 1635, 1697, 1759, 1821, 1883, 1945, 2007, 2069, 2131, 2388, 2645, 2902, 3159, 3416, 3673, 3930}

Table S3. Spin lock powers and offsets used in the $R_{1\rho}$ experiments.

**Nuclei**	**[spin lock power] {offset frequencies}**
	**[ω/2π (Hz)] {Ω_{eff}/2π (Hz)}**
**A_6-DNA 25°C pH 6.8**	
A18-C1'	[150, 200, 250, 300, 400, 500, 600, 700, 900, 1000, 1200, 1400, 1600, 2000, 2500, 3000] {0}<br>[150] {-675, -625, -575, -525, -475, -455, -435, -415, -395, -375, -355, -335, -315, -295, -275, -225, -175, -125, -75, 25, 125, 225, 375, 625}<br>[400] {-1975, -1575, -1375, -1175, -975, -825, -675, -575, -525, -475, -455, -435, -415, -395, -375, -355, -335, -315, -295, -275, -225, -175, -75, 75, 225, 425, 625, 825, 1225, 1625}<br>[600] {-2975, -2575, -2175, -1775, -1375, -1175, -975, -775, -675, -575, -475, -445, -415, -395, -375, -355, -335, -305, -275, -175, -75, 25, 225, 425, 625, 1025, 1425, 1825, 2225, 2625}<br>[1000] {-3375, -2775, -2175, -1575, -1275, -975, -675, -525, -425, -375, -325, -225, -75, 225, 525, 825, 1425, 2025, 2625, 3125}
A19-C1'	[150, 200, 250, 300, 400, 500, 600, 700, 900, 1000, 1200, 1400, 1600, 2000, 2500, 3000] {0}<br>[150] {-675, -625, -575, -525, -475, -455, -435, -415, -395, -375, -355, -335, -315, -295, -275, -225, -175, -125, -75, 25, 125, 225, 375, 625}<br>[400] {-1975, -1575, -1375, -1175, -975, -825, -675, -575, -525, -475, -455, -435, -415, -395, -375, -355, -335, -315, -295, -275, -225, -175, -75, 75, 225, 425, 625, 825, 1225, 1625}<br>[600] {-2975, -2575, -2175, -1775, -1375, -1175, -975, -775, -675, -575, -475, -445, -415, -395, -375, -355, -335, -305, -275, -175, -75, 25, 225, 425, 625, 1025, 1425, 1825, 2225, 2625}<br>[1000] {-3375, -2775, -2175, -1575, -1275, -975, -675, -525, -425, -375, -325, -225, -75, 225, 525, 825, 1425, 2025, 2625, 3125}
A20-C1'	[150, 200, 250, 300, 400, 500, 600, 700, 900, 1000, 1200, 1400, 1600, 2000, 2500, 3000] {0}<br>[150] {-675, -625, -575, -525, -475, -455, -435, -415, -395, -375, -355, -335, -315, -295, -275, -225, -175, -125, -75, 25, 125, 225, 375, 625}<br>[400] {-1975, -1575, -1375, -1175, -975, -825, -675, -575, -525, -475, -455, -435, -415, -395, -375, -355, -335, -315, -295, -275, -225, -175, -75, 75, 225, 425, 625, 825, 1225, 1625}<br>[600] {-2975, -2575, -2175, -1775, -1375, -1175, -975, -775, -675, -575, -475, -445, -415, -395, -375, -355, -335, -305, -275, -175, -75, 25, 225, 425, 625, 1025, 1425, 1825, 2225, 2625}<br>[1000] {-3375, -2775, -2175, -1575, -1275, -975, -675, -525, -425, -375, -325, -225, -75, 225, 525, 825, 1425, 2025, 2625, 3125}

References

Alvey, H.S., Gottardo, F.L., Nikolova, E.N., and Al-Hashimi, H.M. (2014). Widespread transient Hoogsteen base pairs in canonical duplex DNA with variable energetics. Nat Commun *5*, 4786.

Rangadurai, A., Szymaski, E.S., Kimsey, I.J., Shi, H., and Al-Hashimi, H.M. (2019). Characterizing micro-to-millisecond chemical exchange in nucleic acids using off-resonance R1rho relaxation dispersion. Prog Nucl Magn Reson Spectrosc *112-113*, 55-102.

Shi, H., Clay, M.C., Rangadurai, A., Sathyamoorthy, B., Case, D.A., and Al-Hashimi, H.M. (2018). Atomic structures of excited state A-T Hoogsteen base pairs in duplex DNA by combining NMR relaxation dispersion, mutagenesis, and chemical shift calculations. J Biomol NMR *70*, 229-244.
